# Diurnal Changes and Machine Learning Analysis of Perovskite Modules Based on Two Years of Outdoor Monitoring

**DOI:** 10.1021/acsenergylett.4c01943

**Published:** 2024-09-26

**Authors:** Vasiliki Paraskeva, Matthew Norton, Andreas Livera, Andreas Kyprianou, Maria Hadjipanayi, Elias Peraticos, Aranzazu Aguirre, Santhosh Ramesh, Tamara Merckx, Rita Ebner, Tom Aernouts, Anurag Krishna, George E. Georghiou

**Affiliations:** ∇ PV Technology Laboratory, Department of Electrical and Computer Engineering,, University of Cyprus, Nicosia 1678, Cyprus; ‡ PV Technology Laboratory, Department of Mechanical and Manufacturing Engineering, University of Cyprus, Nicosia 1678, Cyprus; § Imec, imo-imomec, Thin Film PV Technology, Thor Park 8320, 3600 Genk, Belgium; ∥ Hasselt University, imo-imomec, Martelarenlaan 42, 3500 Hasselt, Belgium; ⊥ EnergyVille, imo-imomec, Thor Park 8320, 3600 Genk, Belgium; # AIT Austrian Institute of Technology, Center for Energy, Giefingasse 2, 1210 Vienna, Austria

## Abstract

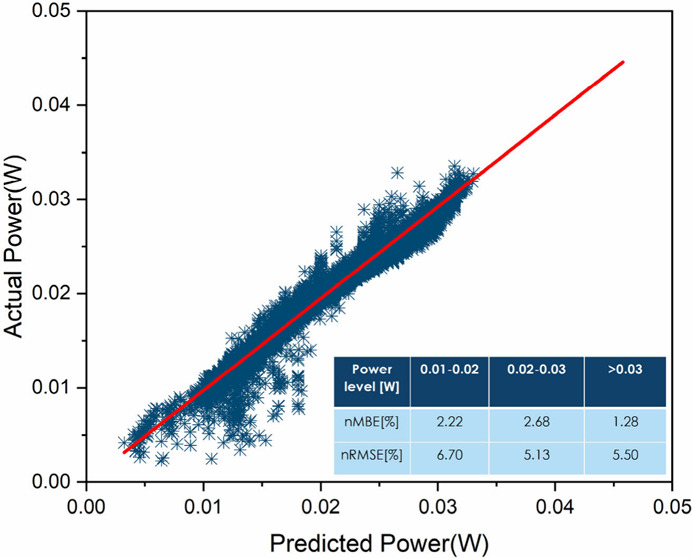

Long-term stability is the primary challenge for the commercialization of perovskite photovoltaics, exacerbated by limited outdoor data and unclear correlations between indoor and outdoor tests. In this study, we report on the outdoor stability testing of perovskite mini-modules conducted over a two-year period. We conducted a detailed analysis of the changes in performance across the day, quantifying both the diurnal degradation and the overnight recovery. Additionally, we employed the XGBoost regression model to forecast the power output. Our statistical analysis of extensive aging data showed that all perovskite configurations tested exhibited diurnal degradation and recovery, maintaining a linear relationship between these phases across all environmental conditions. Our predictive model, focusing on essential environmental parameters, accurately forecasted the power output of mini-modules with a 6.76% nRMSE, indicating its potential to predict the lifetime of perovskite-based devices.

Metal halide perovskites, with their exceptional optoelectronic properties and compatibility with cost-effective large-scale production methods, hold promise as the next-gen photovoltaic (PV) solution. Extensive research over the past decade has boosted perovskite solar cell (PSC) efficiency, reaching certified values of 26% for single junction and 33.7% for perovskite/silicon tandem cells.^1^ Furthermore, lab-made PSCs have passed several aging tests defined by the International Summit on Organic PV Stability (ISOS) protocols.^2^ Despite remarkable progress, challenges such as scalability, long-term stability, and field reliability continue to hinder the commercialization of perovskite-based PV devices.^3^ Several stress factors for the degradation, such as moisture,^4^ UV light exposure,^5^ and temperature,^6^ have been widely reported in the literature. Most of the aging studies in PSCs have been implemented under indoor controlled conditions at low and elevated temperatures, either in constant or cyclic illumination.^7−9^ The metastable behavior of perovskite PV is a complicated phenomenon in a historical context. While some studies report performance recovery in the dark and loss in perovskite device performance under light exposure,^9,10^ some other studies report the opposite trend: performance degradation in the dark and restoration under light.^11^ Furthermore, these two opposite trends were observed for the same devices at various degradation stages.^12^ Moreover, the processes involved in both reversible and irreversible degradation were found to be sensitive to varied stoichiometries^13^ and dependent on the bias conditions of the device under test.^14^ Reversible or temporary changes of perovskite devices observed indoors raise questions about the actual extent of degradation when operating in real outdoor environments.^15^

So far, a number of studies have been conducted on outdoor performance monitoring of perovskite-based devices.^16,17^ Outdoor stability testing has been reported for different types of perovskite devices, including single-junction cells,^18,19^ modules,^16,20^ and tandem cells.^21,22^ Recently, 2-year outdoor data and 10-month outdoor data of perovskite cells have been presented^23,24^ but without the investigation of the impact of metastability effects on a daily basis. Diurnal changes of PCE values of carbon-based perovskite cells in a short-term outdoor exposure were investigated by De Rossi et al.^25^ Most papers that refer to outdoor module measurements^26−28^ refer to a limited period of testing (<1 year) and do not investigate the diurnal metastable behavior of perovskites but rather the impact of temperature and spectrum in combination with life cycle assessment studies. Qualitative and quantitative investigation of the effect of light soaking effects on the metastable behavior of electrical parameters on a daily basis was presented recently by Remec et al.^29^ However, this investigation does not extend over different degradation stages of the samples outdoors. An attempt to correlate the magnitude of nighttime degradation or recovery with performance recovery or degradation upon illumination for any major electrical parameter is absent in the literature now. Finally, the investigation of evolution of the electrical parameters outdoors during nighttime in the field is absent from all of the above publications.

Machine learning is used to analyze large amounts of data and can be used as a powerful tool to evaluate PSCs and thus optimize their performance.^30−32^ Studies have shown that it can accurately predict the power conversion efficiency of PSCs based on composition and structural parameters.^33,34^ Machine learning algorithms have been utilized before^35^ using indoor stability data sets to predict the outdoor stability of perovskite-based devices. However, machine learning models using high-throughput outdoor stability data to predict the time-series power output are still limited in the literature. To this end, understanding the diurnal degradation and overnight recovery under real outdoor environmental conditions, along with developing a machine-learning-based predictive model using outdoor stability data, will accelerate the advancement of perovskite photovoltaic technology.

This study presents the outdoor stability of perovskite mini-modules over a two-year period, with an emphasis on the evolution of key electrical parameters. We conducted a detailed statistical analysis of current–voltage (I–V) curves from different perovskite device configurations tested in outdoor conditions and tried to correlate the diurnal and nighttime changes in the perovskite devices. This analysis revealed patterns of performance degradation during daylight hours and overnight recovery. We also explored changes in electrical parameters such as current, voltage, and fill factor across day and night cycles to gain insights into the impact of irradiance and temperature on the electrical performance of these devices, as well as the underlying mechanisms responsible for their diurnal fluctuations. Additionally, we developed a data-driven predictive model using the eXtreme Gradient Boosting (XGBoost) regression technique. This model predicts the output power time series of perovskite modules, incorporating major input parameters that affect the power output while excluding diurnal variables.

In this study, we employ perovskite mini-modules with the p-i-n device architecture glass/ITO/hole transport layer (HTL)/perovskite/electron transport layer (ETL)/ITO. The chosen perovskite composition for this research is FA_0.8_Cs_0.2_Pb(I_0.94_Br_0.06_)_3_. For the HTL, sputtered NiO is utilized, while the electron ETL is varied between two configurations: LiF/C60/BCP and LiF/C60/LiF. All test samples were laminated using the same material to ensure uniformity in environmental protection. This architecture of mini-modules has been previously reported to show high stability under indoor accelerated testing.^36^ We deployed these mini-modules for outdoor testing in a fixed plane array situated in Nicosia, Cyprus, from the summer of 2021 to the summer of 2023. Throughout this period, I–V measurements were consistently gathered over several months to evaluate the performance. A comprehensive breakdown of the sample characteristics, and configurations is detailed in Table 1of Table 1 of Supplementary Discussion 1. The outdoor performance data discussed in this work pertain to the exposure period detailed in Table 1, which also summarizes the initial electrical characteristics of the samples under standard testing conditions.

**Table 1:** Summary of the Major Structural and (Initial) Electrical Characteristics in the Perovskite Samples Studied in This Work

Mini Module ID	Exposure period	Perovskite Absorber	Electron Transport Layer (ETL)	Hole Transport Layer (HTL)	Isc (mA)	Voc (V)	FF (norm.u)	PCE (%)
**ETL1_ A**	22/07/2021–22/07/2023	FA_0.8_Cs_0.2_Pb(I_0.94_Br_0.06_)_3_	LiF/C60/BCP	NiO	13.81	7.11	0.56	13.38
**ETL2_A**	11/08/2022–11/08/2023	FA_0.8_Cs_0.2_Pb(I_0.94_Br_0.06_)_3_	LiF/C60/LiF	NiO	11.52	6.88	0.65	14.32
ETL2_B	11/08/2022- 11/08/2023	FA_0.8_Cs_0.2_Pb(I_0.94_Br_0.06_)_3_	LiF/C60/LiF	NiO	12.52	6.79	0.68	14.46

The normalized daily PCE values of the different perovskite mini-modules were calculated using daily averages of reverse I–V sweep parameters and plane-of-array pyranometer irradiance. The installation date and the operating conditions for each sample can be found in Supplementary Discussion 2. Supplementary Discussion 3 shows the temporal profile of both the electrical and environmental parameters of the modules for selected clear sky days.

Figure 1(a) shows that the different samples developed roughly the same PCE reduction over the first year of testing. To further evaluate the performance of the modules, the daily average performance ratio was measured on two representative samples, one for each configuration, over a period of one year. The performance ratio (PR) time series for the ETL1_A sample was calculated for the period July 2021–July 2022 while for the ETL2_B sample it was calculated for the period August 2022–August 2023 using the field measurements of the array DC power (Pmax) and in-plane irradiance. The PR indicates the overall effect of losses on the array’s rated output and is defined as the ratio of the array yield (YA) and the reference yield (Yr). Array yield is the sum of the array energy divided by the nominal power of the test PV array, while the reference yield is the sum of global irradiation at the plane of the array divided by the global irradiance under standard test conditions. Essentially, the PR describes the relationship between the actual/measured output and the theoretical/expected PV energy output for a given reporting period based on the system name-plate rating. Figures 1(b) and 1(c) show the computed performance ratio (PR) for ETL1_A and ETL2_B, respectively. The PR results for ETL2_A presented a larger dispersion and thus were not taken into consideration in this analysis. It is evident that both samples exhibit an initial burn-in period, followed by a period of relatively constant PR. During the initial burn-in period, the decrease in PR appears linear, prompting the fitting of a bilinear curve. The bilinear curve consists of two lines intersecting at the point of the slope change. Three crucial estimated parameters simultaneously determine the optimal bilinear curve: the slopes of the two lines comprising the bilinear curve and the day of intersection between these lines. The slopes of the two lines correspond to the performance loss rate (PLR) of the sample at each corresponding period. The mathematical expression of this curve and the error minimized to achieve the fitting of the curve are described in Supplementary Discussion 4, while details of this algorithm are provided in ref (37).

**Figure 1:**
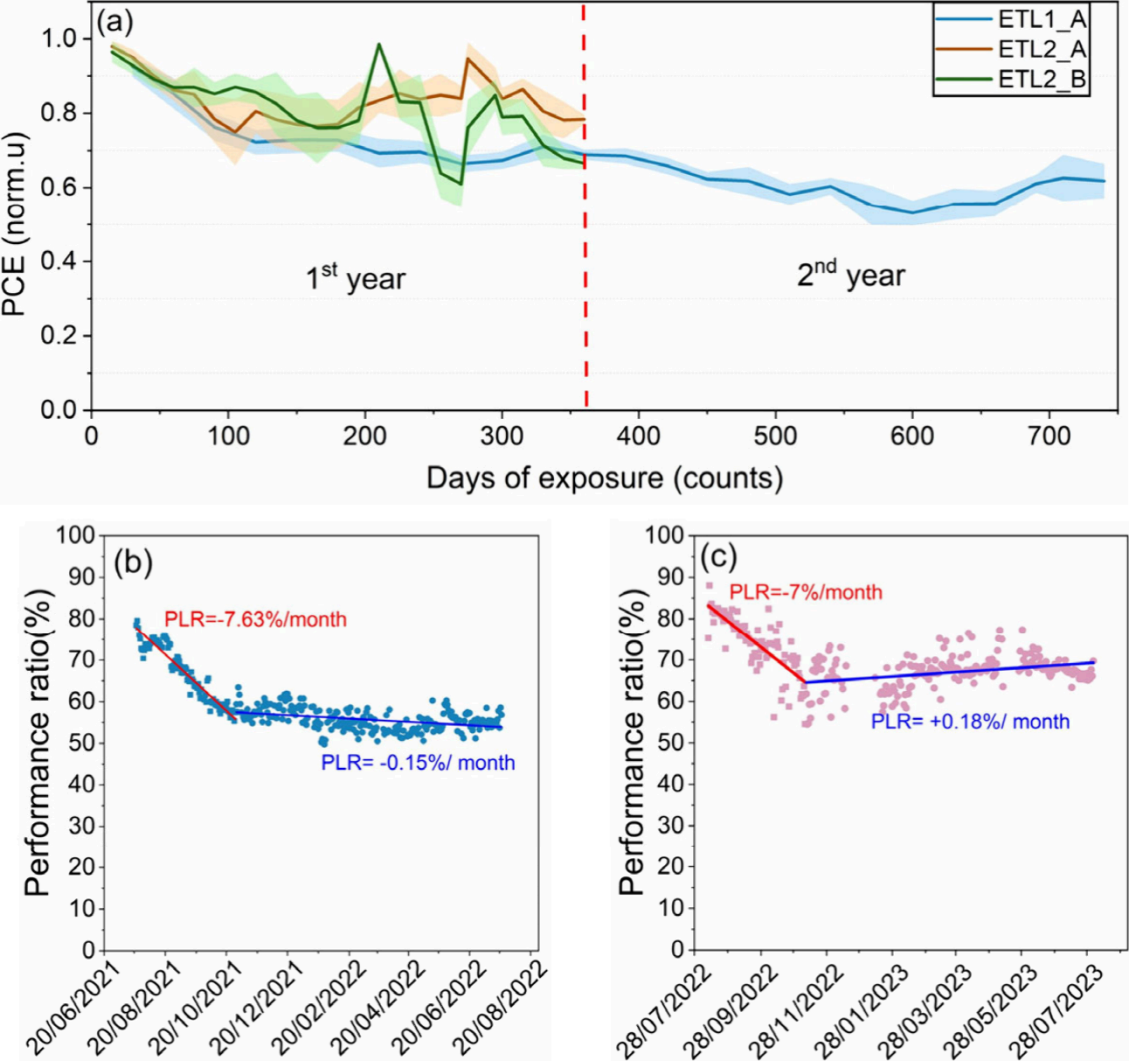
Long-term performance and performance loss rate. (a) Daily average power conversion efficiency (PCE) in normalized units from perovskite mini-modules of different structures. The initial PCE is shown in Table 1. The lifetime of the samples under test ranges between some months and up to two years of testing. Efficiency at reverse sweeps is reported in the graph. (b and c) Performance ratio evolution over time for (b) ETL1_A and (c) ETL2_B samples. The performance loss rate was estimated with a differential evolution algorithm on the performance ratio time series.

Figure 1(b) shows a PLR for the ETL1_A sample of −22.9% during the initial burn-in period and −1.34% during the period of relatively constant PR. This equates to PLR values of −7.63% and −0.15% per month, respectively. The estimated intersection point occurs around the 91st day (∼3 months). Similarly, Figure 1(c) shows that the ETL2_B sample presented a PLR of −21% during the burn-in period and +1.7% over the period of relatively constant PR. This equates to PLR values of −7% and +0.18% per month, respectively. The estimated day of intersection remains at the 91st day. To look at the root causes of the PCE degradation of the modules, Isc, Voc, and FF were also analyzed over time (Supplementary Discussion 5). Over the first month of testing, current reduction was the major origin of performance degradation in all samples (Figure 10, Supplementary Discussion 5) and is likely to be related to ionic movement, since ionic space charges can be the origin of transient current loss in PSCs.^38^ Some samples also showed an increase in open-circuit voltage over the first month of testing, which can be attributed to metastability or light-soaking effects present in these devices. FF reduction over the period was also observed for all samples and might be attributed to interface defects between the hole transport and absorber layers.^39^

During long-term outdoor testing, the bias load condition of some samples between I–V curves was changed from *V*_oc_ load to MPP and vice versa. This was done to explore the changes that might occur in the evolution of electrical parameters, including diurnal PCE degradation and recovery. The details of each bias state are provided in Table 2, Supplementary Discussion 2. The most important observation arising from this is that the bias load transition from *V*_oc_ to the MPP bias load between IV scans induced a reduction in the Voc values of samples ETL1_A and ETL2_A. Figure 2 demonstrates a more pronounced reduction of the Voc values with the load transition in the ETL2_A sample. In the case of the ETL1_A sample, the Voc value reduction can be observed at the fourth, fifth, and 11th month of testing when the bias load switched from Voc load to MPP load, and similarly in the case of the ETL2_A sample between the fifth and eighth month of testing for the same reasons (see Supplementary Discussion 5). One likely explanation for this is that different distributions of charged species arise in the devices due to the presence of different electric bias.^40^ The ion distribution at open circuit conditions and under bias at MPP will be different, leading to different electrical performance thereafter (Supplementary Discussion 5).

**Figure 2:**
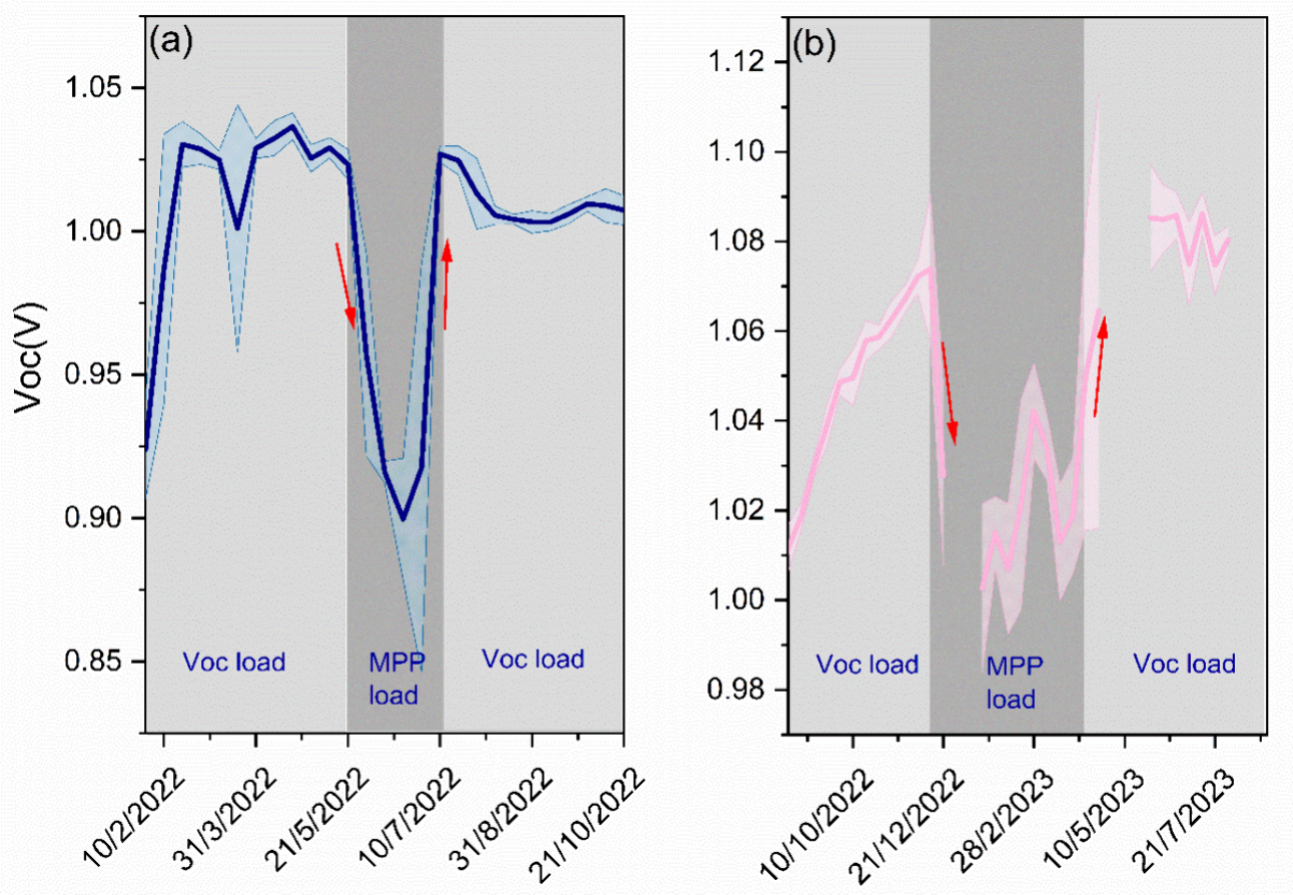
Open-circuit voltage value evolution over time for the samples (a) ETL1_A and (b) ETL2_A. The reduction of the open-circuit voltage values of the perovskite devices with the transition from the Voc load to MPP load between IV sweeps is indicated by the arrows in both cases.

To gain a deeper understanding of the diurnal changes occurring in the perovskite modules, the diurnal performance degradation (DPD) and the diurnal performance recovery (DPR) overnight were studied. The diurnal performance degradation (DPD) corresponds to the difference in performance values at the beginning and the end of each day outdoors at the same irradiance levels. The beginning and the end of the day were selected to correspond to irradiances in the morning and afternoon close to 400 W/m^2^. The values were then normalized to the initial efficiency value of the first day to have comparable and normalized results from all days in the field. Similarly, the diurnal performance recovery (DPR) values were calculated by considering the difference between the final efficiency value of the previous day and the initial efficiency value of the next day at the same irradiance levels (400 W/m^2^). The resulting efficiency recovery values were normalized for comparative purposes. The equation and the points utilized for diurnal performance degradation and diurnal performance recovery overnight can be found in eqs 1 and 2 and Figure 3.
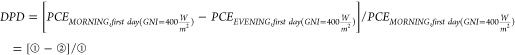
1
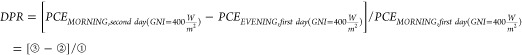
2

**Figure 3:**
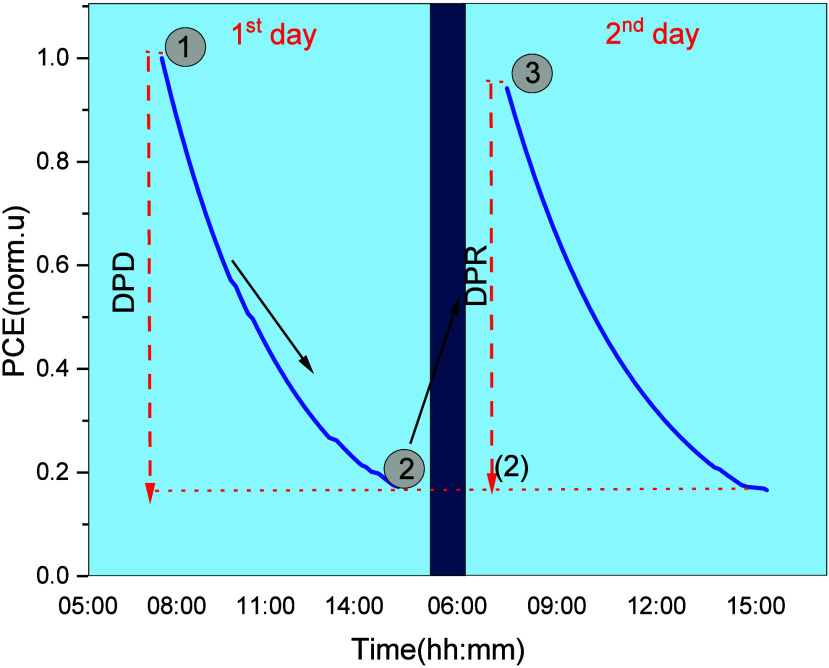
Representation of a diurnal change in performance for one module under test for two consecutive days. The points used to extract diurnal performance degradation and diurnal performance recovery overnight are indicated in the schematic.

DPD and DPR were calculated to establish the typical values of diurnal changes and to extract the degradation-to-recovery ratio and its seasonal dependence. A flowchart of the procedure followed to extract the DPD and the DPR overnight is provided in detail in Supplementary Discussion 6. Figure 4(a) and Figure 4(b) show the normalized DPD and DPR overnight from representative modules of each perovskite architecture. The DPD and DPR from ETL2_B are provided in Supplementary Discussion 7. DPD and DPR were recorded mainly in the range of 0–25% at all modules under test and were unrelated to the degradation stage. Larger dispersions of these values are apparent in months with fewer data points due to either site maintenance outages or environmental conditions. (December 2022–January 2023, May 2023).

**Figure 4:**
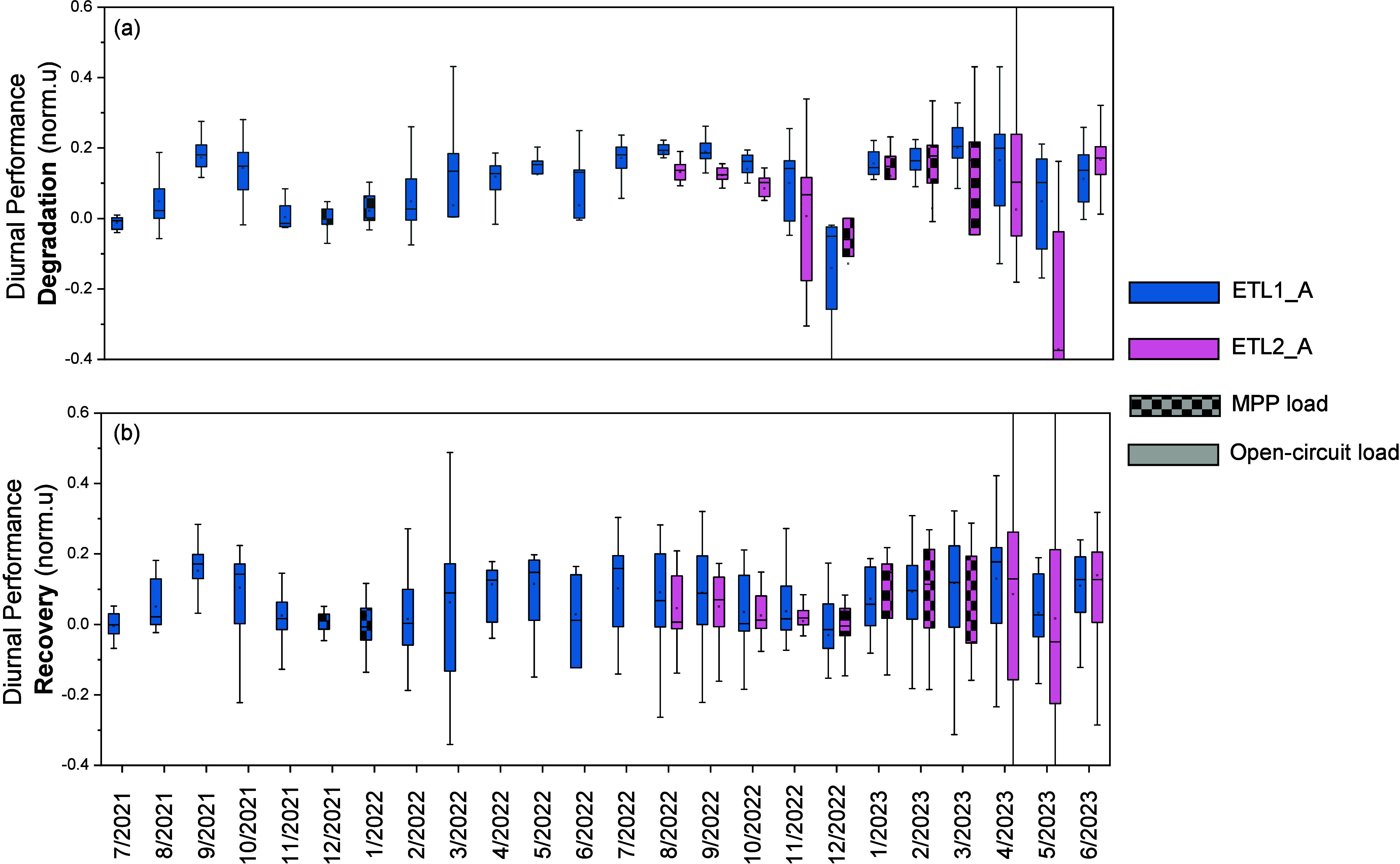
(a) Diurnal performance degradation and (b) diurnal performance recovery overnight for mini-modules, ETL1_A and ETL2_A. Open-circuit load was utilized mainly during the outdoor testing of modules, while MPP load was applied only in some months of testing. The boxplot results take into consideration the mean and median values of the data collected from each day in the field within each month of testing.

The DPD of the ETL1 series sample was analyzed in relation to the temperature and irradiance levels. Boxplots of the DPD at different temperature levels from the sample ETL1_A, which presented the longer lifetime, are shown in Figure 5. A larger statistical data set was available for the plot of the ETL1_A sample since this was tested for two years outdoors compared to the ETL2 series samples which exhibited statistical data from roughly a year of testing. DPD values were studied in the range 20 °C–60 °C with a temperature step of 10 °C. Normalized values for the diurnal parameters were utilized. The ETL1_A sample presented a slight increase in DPD and DPR at higher temperature levels. A statistically significant difference was found between the results at low and high temperatures (between the results in bins [20,40] and [50,60]). Mobile ion migration and accumulation are dramatically affected by thermal effects, with more freely moving ion generation present at higher temperature levels,^41^ which may result in larger diurnal changes. To support these findings, two identical modules with the ETL1 configuration were tested indoors under a high throughput aging system to study the impact of temperature on DPD and DPR. The aging system cycled day-night irradiance levels at different temperatures to mimic outdoor conditions during the summer and winter. The results showed that higher temperature levels did lead to higher DPD, which agrees with the outdoor results. Details of this experiment are provided in Supplementary Discussion 8. Lastly, no trend of the diurnal values with irradiance was detected in the ETL1_A sample, suggesting that irradiance might not play a role in the DPD and DPR values (Figure 5).

**Figure 5:**
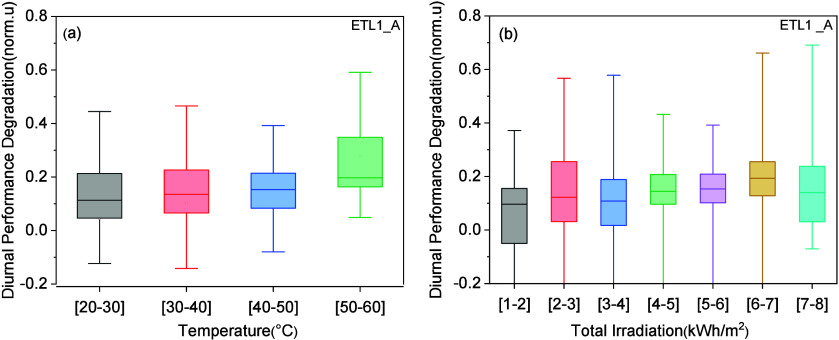
Diurnal performance degradation for the ETL1_A sample at different temperatures and irradiance levels.

A statistical analysis was applied to the diurnal data for all samples tested outdoors (Supplementary Discussion 9). The normalized DPD and DPR were separated into bins of 5% and the frequency of occurrence at each bin was calculated for each module. Long-lasting modules experienced degradation and recovery mostly in the 0%–5% and 15%–20% bins. The DPD outdoors is determined by the deviation of the output power values in the morning and evening hours (Supplementary Discussion 10). The diurnal degradation and recovery of the maximum power point current (I_mp_) and maximum power point voltage (V_mp_) were also investigated to uncover the main origin of the diurnal changes. Based on the results shown in Supplementary Discussion 11, diurnal I_mp_ degradation occurs on most days of outdoor exposure for all samples, in line with the DPD behavior. On the other hand, the V_mp_ presented an increase over the day and a reduction overnight due to light-soaking effects. It is worth noting that since V_mp_ values are strongly influenced by temperature, the diurnal V_mp_ degradation was calculated by restricting the temperature difference between morning and evening to within 5 °C in the DPD algorithm (Supplementary Discussion 6). However, the results remain the same again. The diurnal increase of Vmp was found to be lower than 10% at all samples under tests and for most days of testing.

The diurnal changes in electrical parameters from sample ETL2_A for one representative day in autumn 2022 are summarized in Figure 6. The diurnal changes of the major electrical parameters of sample ETL2_B for the same day in Autumn 2022 are shown in Figure 23 (Supplementary Discussion 11). Based on the above, the DPD and DPR processes are mainly driven by current, not voltage. Ion migration affects the current in perovskite materials, and for this reason, changes in I_mp_ seem to determine both the DPD and DPR in perovskite samples. After two years of testing, the ETL1 _A module was kept indoors for 1 week and then exposed outdoors again (Supplementary Discussion 12) to study the recovery dynamics during dark storage. The major path of output power recovery originated from the current and not from the voltage, in agreement with the outdoor diurnal data results.

**Figure 6:**
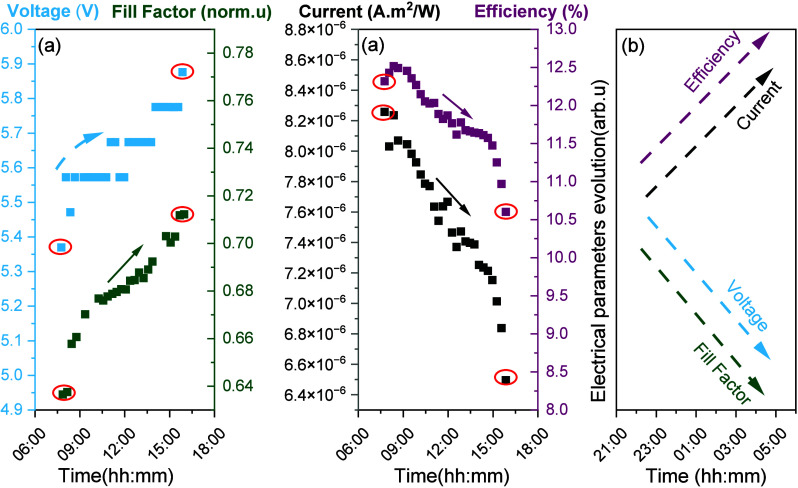
Diurnal changes occur at all major electrical parameters of a perovskite device during (a) day and (b) night hours. Current and voltage values correspond to the ones at maximum power point conditions (Imp, Vmp). Data correspond to the 38th day of exposure of sample ETL2_A (where PCE degradation was around 9%) and at irradiances higher than 400 W/m^2^. The data in the red circles have been utilized for calculating the diurnal degradation and recovery values for PCE, Imp, Vmp and FF. Data corresponds to a day with high irradiation and temperature levels.

Based on the analysis of diurnal FF degradation from long-term outdoor testing of modules (see Supplementary Discussion 11), it was found that the FF increases during the day. We hypothesize that the device’s morning performance is primarily affected by mobile defects. During the preceding dark period, defect accumulation occurs due to the built-in potential. Upon initial exposure to light, this accumulation begins to decrease. As light intensity increases, the net electric field under open-circuit conditions shifts direction. This shift drives accumulated ions away from the interface, reducing interface recombination and enhancing charge extraction, which improves both the open-circuit voltage (Voc) and fill factor (FF). However, as the day progresses, a new degradation pathway emerges due to the evolving distribution of mobile defects, ultimately diminishing performance. The observed performance changes suggest that multiple degradation pathways are activated throughout the day. At this stage, we cannot precisely identify the underlying mechanisms from the current data. Further characterization and modeling will be necessary to confirm these phenomena, which is our next step.

The DPD-to-DPR ratio was analyzed to study the interdependence of these qualities and any fluctuations present due to seasonal effects. The ratio was found to be broadly consistent across all temperature and irradiance levels and degradation stages (see Figure 7). This result holds for all samples under test even if absolute values of DPD and DPR are taken into consideration (Supplementary Discussion 13). However, the exact value of the DPD-to-DPR ratio depends on the specific perovskite structure and sample. The value of the slope varies from device to device, with lower values obtained for samples that degrade more rapidly. Lastly, the linear correlation between diurnal degradation and recovery was observed for the maximum power point current (Imp) and performance (PCE) and it was not the case for the maximum power point voltage (Vmp) and Fill Factor (FF) (Figure 27, Supplementary Discussion 14).

**Figure 7:**
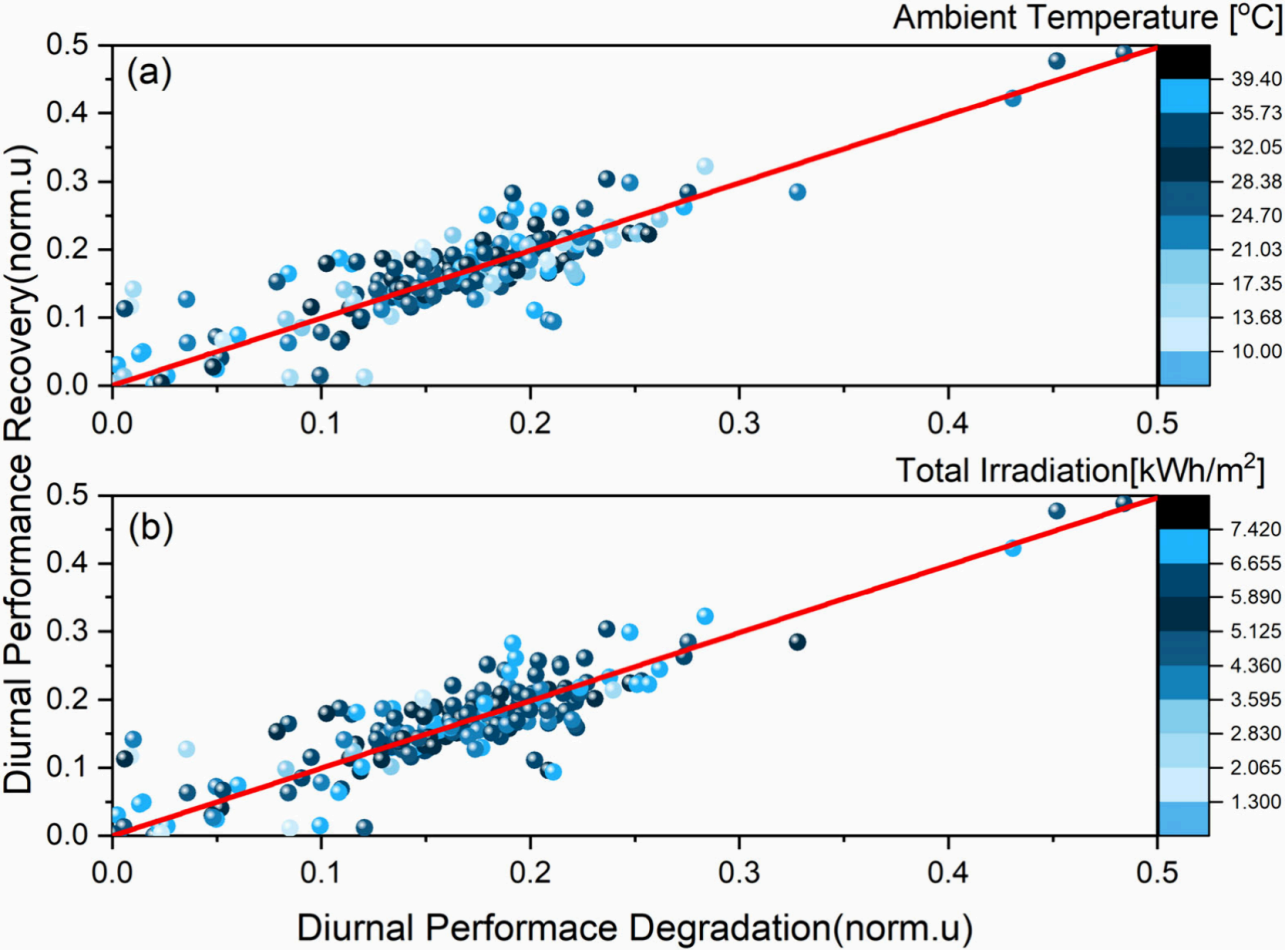
Diurnal performance recovery overnight against diurnal performance degradation at (a) different ambient temperatures and (b) total irradiation levels during the two years of outdoor testing. Data correspond to the ETL1_A sample, which presented a longer lifetime.

Finally, a data-driven predictive model was constructed to simulate the PV performance using the exponential gradient boosting (XGBoost) regression model. The approach for the development of the predictive model relies on optimized model hyperparameters by following structured supervised learning regimes that utilize low partitions of historic data and input features for the training procedure. The constructed XGBoost regressor was used to predict the PV power output (*P*_max_), since it has exhibited high-performance accuracies in prediction problems.^42^ The XGBoost is an ensemble algorithm that combines several decision trees using the boosting method to generate the desired output prediction. Once decision trees were trained, ensemble modeling by weighted averaging was performed to pool the results from multiple trees and average them using weights based on accuracy to minimize the error. The core of XGBoost is to optimize the objective function’s value by using gradient descent to create new trees based on the residual errors of previous trees. The equations used in the photovoltaic system machine learning predictive model are provided in Supplementary Discussion 14. Initially, the XGBoost regression model was implemented using the available essential measurement parameters such as module temperature, normal irradiance at the plane of array, and output power. The PV time series was separated into training and test subsets. More specifically, a 70:30% training and test set approach was followed. The train set (70% using random samples) was used for the model’s training process (developing the model) and included the dates between July 2021 and June 2022, while the test set (30%) was used for assessing the model’s performance and included dates between June 2022 and April 2023. Comparison of the measured and predicted power output for four (4) selected days in the field can be found in Figure 8. Better agreement between the predicted and real power is detected during clear sky days.

**Figure 8:**
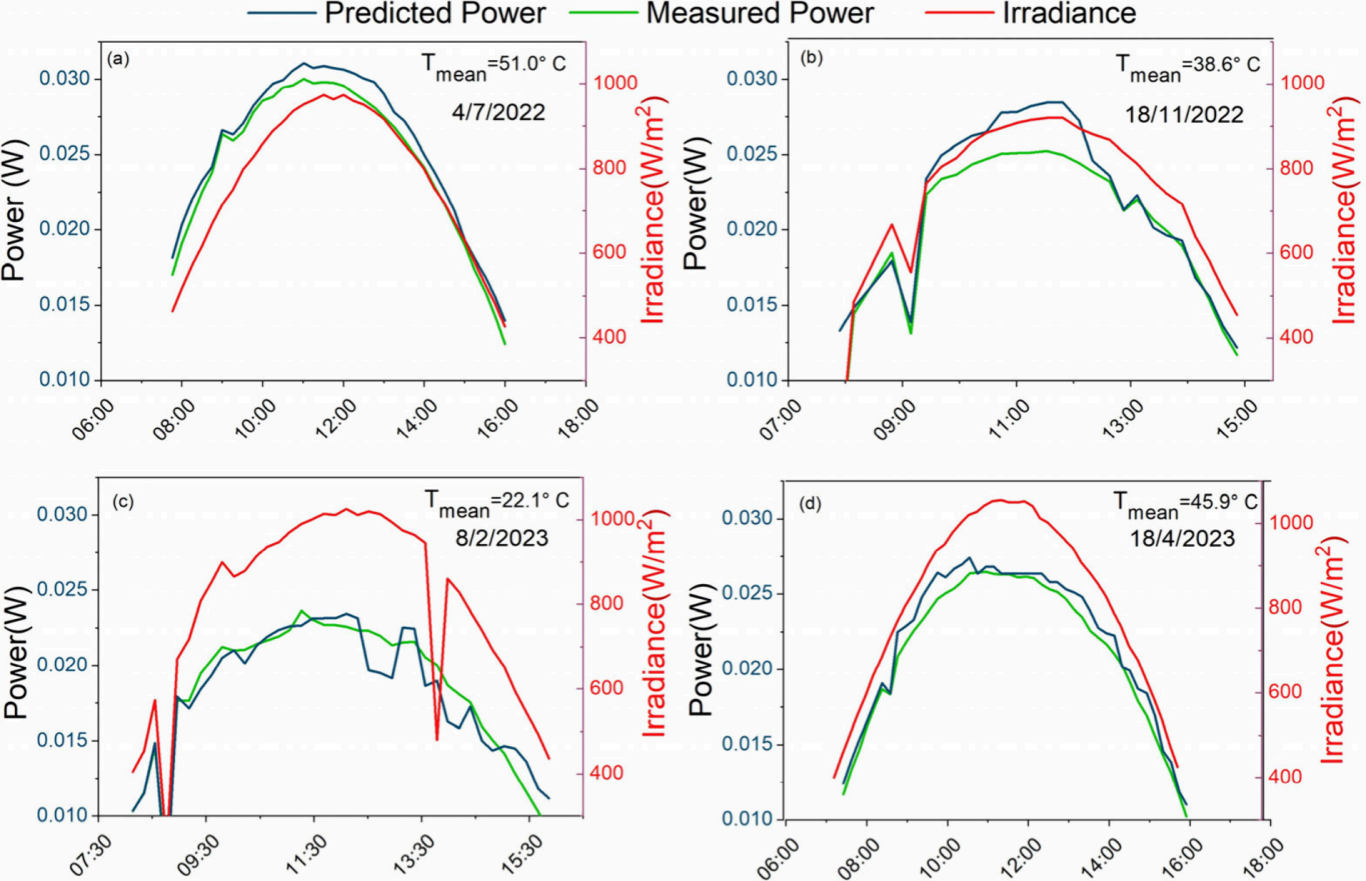
Measured and predicted power output from sample ETL1_A for four (4) selected days in (a) July 2022, (b) November 2022, (c) February 2023 and (d) April 2023. The mean module temperature for each day is provided in each graph.

To evaluate the predictive accuracy of the constructed model, the normalized root-mean-square error (nRMSE) and the mean bias error (MBE) metric were used.^43,44^ The root-mean-square error considers the square of the difference between real and predicted data to signal large errors in the predictions. The mean bias error (MBE) operates differently by adding the standard difference and indicates whether the predictions are under- or overestimated. These metrics can be normalized by considering the average of the actual values, yielding the normalized root-mean-square error (nRMSE) and the normalized Mean Bias Error (nMBE).

A linear behavior was found between the actual and predicted power at different temperatures and irradiance levels. The results are summarized in Figure 9. The proposed model achieved a maximum nRMSE of 6.70% when applied to the test set data set at low power levels (0.01 < power < 0.02), giving evidence of large discrepancies between the actual and predicted power at lower power levels. At higher power levels (>0.02 W) the nRMSE lies at lower values, giving evidence of better prediction ability of the regression model at high output power of the PV module. The nRMSE and MBE metrics are both demonstrated in the inset of Figure 9. Based on the modeling results, it seems that the essential input parameters such as temperature, irradiance, and measured power in the regression model are sufficient to predict the power time series of perovskites.

**Figure 9:**
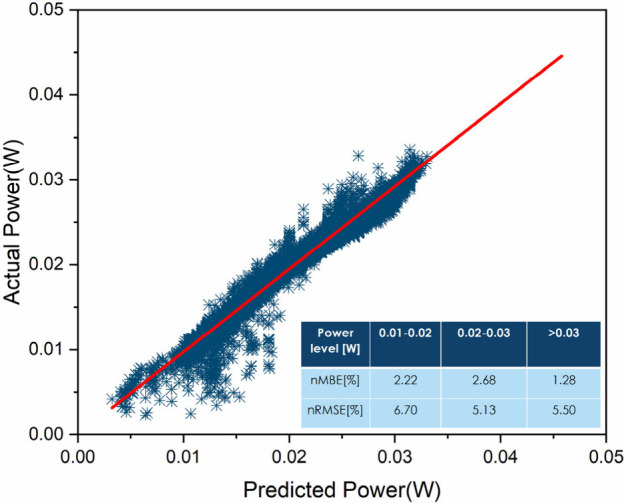
Actual vs predicted power for perovskite mini-module ETL1_A Module using the eXtreme Gradient Boosting (XGBoost) regression model. The normalized root-mean-square error (nRMSE) and the normalized mean bias error (nMBE) metrics are provided in the inset.

In summary, a long-term outdoor study was conducted on perovskite mini-modules in two configurations. The most durable mini-module maintained 78% of its initial PCE after one year. Performance loss rates during the burn-in period of perovskites were found to be around 7%–8% per month for the perovskite configurations tested. Long-term I–V monitoring facilitated the analysis of diurnal performance changes, including variations in current, voltage, and fill factor. Our high-level statistical analysis revealed a consistent pattern: diurnal performance degradation was invariably followed by overnight recovery, regardless of the stage of degradation or irradiance and temperature. The linear correlation between diurnal degradation and recovery was observed for the maximum power point current values. Statistical data further indicated a decrease in diurnal performance and current throughout the day, with recovery occurring overnight. Conversely, the voltage and fill factor tended to improve during the day but decreased overnight. This behavior is generally observed and is independent of the stage of degradation or environmental variables such as irradiance and temperature. We also implemented a data-driven predictive model using the XGBoost regression to forecast the power output. The model achieved a normalized nRMSE of 6.76% on the test set, demonstrating a strong correlation between the actual and predicted power. Notably, the accuracy of predictions was higher at higher power and irradiance levels, with greater deviation at lower power values. Our findings suggest that machine learning models effectively predict the outdoor behavior of perovskites. We believe that this work will serve as a crucial stepping stone in advancing our understanding of degradation features when perovskite solar cells are tested under real outdoor conditions.

## Materials and Methods

### Materials

Indium tin oxide (ITO) coated glass substrates were purchased from Colorado Concept Coatings LLC. Formamidinium bromide (FABr) and formamidinium iodide (FAI) were purchased from Greatcell Solar Materials. Lead iodide (PbI2) and cesium iodide (CsI) was purchased from Tokyo Chemical Industry (TCI). Fullerene C60 was purchased from Nano-C. 2,9-Dimethyl-4,7-diphenyl1,10-phenanthroline (BCP) was purchased from Luminescence Technology Corp. (Lumtec). Lithium fluoride (LiF), anhydrous dimethylformamide (DMF), and anhydrous 1-methyl-2-pyrrolidone (NMP) were purchased from Sigma-Aldrich. Absolute acetone and isopropyl alcohol were purchased from VWR. All materials were used as received.

### Perovskite Precursor Solution

For the preparation of 1 mL of solution, we dissolve 38.8 mg of CsI, 81.9 mg of FAI, 15 mg of FABr, 342.7 mg of PbI2 in 0.950 mL of DMF, and 0.05 mL of NMP. We stir the solution at room temperature until all powder dissolves.

### Device Fabrication

A set of perovskite solar modules with an aperture area of 4 cm^2^ was fabricated for investigation. These modules were deposited onto 3 × 3 cm^2^ glass substrates.

P1 scribe was performed on the ITO-coated glass substrates, which isolate the ITO layer. Then the ITO-coated glass substrates were subsequently cleaned for 5 min in an ultrasonic bath of detergent, deionized water, acetone, and isopropanol. The substrates were transferred to an N_2_ filled glovebox for the deposition of HTL NiOx. The 15 nm NiOx was deposited by DC sputtering. After the sputtering, the NiOx was annealed at 300 °C for 20 min in air. The NiOx substrates were transferred to a nitrogen-filled glovebox, where the perovskite layer was deposited. A blade coater with an air knife for N_2_ gas quenching attached to it was used for the perovskite coating. A uniform layer was obtained by using a coating speed of 2.5 mm/s. After the coating, gas quenching via an air knife was performed. Finally, the perovskite layer was annealed at 100 °C for 30 min. After perovskite layer deposition, the samples were transferred into a high-vacuum chamber (base pressure ∼10–8 Torr) to deposit ETL by thermal evaporation. Two different ETLs were considered in this work. ETL1 consisted of a trilayer of LiF (0.8 nm), C60 (40 nm), and BCP (5 nm), referred to as LiF/C60/BCP, which was evaporated within a thermal chamber. ETL2 consisted of a trilayer of LiF (0.8 nm)/C60 (20 nm)/LiF (0.8 nm). P2 laser scribe was performed by a picosecond UV laser. Then, 70 nm thick ITO was sputtered. After this, finally, the P3 scribing was performed with a UV laser. After this step, the modules were laminated. The Sn-coated Cu busbars, which were 50 μm thick, were attached by using conductive glue (Nagase Chemicals, DB-1588-4). The lamination package included a polyolefin encapsulant (Borealis, BPO8828F) and two 2-mm-thick soda lime glass plates, serving as the front and back sheets. The edges were sealed by using butyl rubber (Quanex, Solargain LP-03). The lamination was performed at 130 °C for 15 min at 1 atm pressure over the sample. More details regarding the module architecture can be found in Supplementary Discussion 15.

### Aging of Perovskite Mini-Modules

The three (3) perovskite samples under study have been aged outdoors at different time periods between July 2021 and July 2023 at the FOSS testing site at a fixed plane array, and a data acquisition system was utilized to collect the I–V curves from the mini-modules. The electrical measurements have been acquired by a single current–voltage source-meter multiplexed to take sequential measurements from the devices under test. The test laboratory facilities consist of 14 channels connected to relay panels, the required electrical equipment (national instruments PC, relay control board, Keithley 2430 source meter) and a weather station. LabVIEW software was designed to record the current–voltage (I–V) traces every 10–15 min at high Global Normal Irradiance (GNI) conditions (GNI > 400 W/m^2^). The I–V curves were acquired as quickly as possible to keep the irradiance constant and avoid sudden changes due to clouds (sweep rate 0.5V/sec was applied). The sweep rate and the measurement frequency determined the number of I–V curves collected per hour. Both forward (<0 V to > open-circuit voltage (Voc)) and reverse (>Voc to <0 V) voltage sweeps have been applied to the devices. The forward-first voltage approach has been used at almost all instances. The system permits the application of different bias load between I–V scans. For the measurements shown in this report, open-circuit load was mainly applied between I–V scans. However, at some months of testing and in some samples under test, MPP load was applied. The MPP load did not employ active tracking; rather, the system was designed to hold the terminal voltage of the device under test at the average maximum power point voltage of both the forward and reverse I–V sweeps. Thus, the MPP voltage was updated immediately after an I–V sweep and held constant until the next I–V sweep occurred. At low irradiance levels (<350 W/m^2^) and during the night period, the samples were kept at open-circuit conditions. The detailed description of the outdoor infrastructure setup utilized for performance monitoring of samples can be found in Supplementary Discussion 16.

Environmental data such as Global Normal Irradiance (GNI), Direct Normal Irradiance (DNI), wind speed, relative humidity, pressure, ambient temperature, and module temperature were collected simultaneously with the electrical characteristics of the samples using a real-time data acquisition system (see Supplementary Discussions 16 and 17). The ambient and module temperatures were measured simultaneously with I–V curve collection and at irradiances higher than 400 W/m^2^. The instrument used for the collection of the global normal irradiance was a first-class pyranometer Hukseflux SR 11 with uncertainty ±2%. The operating temperature range of the sensor is between −40 and 80 °C, while the spectral range is between 285–3000 nm. The sensor for measuring the ambient temperature is a Rotronic HC2S3 with uncertainty ±0.1 °C at 23 °C. The module temperature was measured with a PT 1000 resistance thermometer located at the back of the module under test. PT 1000 measures at the temperature range from −50 to 500 °C. The humidity was measured with a Rotronic HC2S3 probe with uncertainty ±0.8 RH% at 23 °C. The environmental conditions at the tested location belong to Csa conditions according to the Köppen climate classification.

## Supporting Information Available

Supplementary Discussion 1–17, Figures 1–35, and Tables 1–5, as mentioned in the text. (PDF)

## References

[ref1] NREL. Best Research-Cell Efficiency Chart, Photovoltaic Research. https://www.nrel.gov/pv/cell-efficiency.html, Accessed 13/09/2024.

[ref2] Khenkin M. V.; et al. Consensus statement for stability assessment and reporting for perovskite photovoltaics based on ISOS procedures. Nat. Energy 2020, 5 (1), 35–49. 10.1038/s41560-019-0529-5.

[ref3] Duan L.; et al. Stability challenges for the commercialization of perovskite–silicon tandem solar cells. Nat. Rev. Mater. 2023, 8 (4), 261–281. 10.1038/s41578-022-00521-1.

[ref4] Mishra A. K.; Shukla R. K. Effect of humidity in the perovskite solar cell. Mater. Today Proc. 2020, 29 (3), 836–838. 10.1016/j.matpr.2020.04.872.

[ref5] Domanski K.; Alharbi E. A.; Hagfeldt A. Systematic investigation of the impact of operation conditions on the degradation behaviour of perovskite solar cells. Nat. Energy 2018, 3, 61–67. 10.1038/s41560-017-0060-5.

[ref6] Zhang H.; Qiao X.; Shen Y.; Wang M. Effect of temperature on the efficiency of organometallic perovskite solar cells. J. Energy Chem. 2015, 24 (6), 729–735. 10.1016/j.jechem.2015.10.007.

[ref7] Tayagaki T.; Kobayashi H.; Yamamoto K.; Murakami T. N.; Yoshita M. Ultraviolet-light–dark cycle analysis of degradation in perovskite solar cells. Sol. Energy Mater. Sol. Cells 2023, 263, 112583 10.1016/j.solmat.2023.112583.

[ref8] He J.; et al. Influence of phase transition on stability of perovskite solar cells under thermal cycling conditions. Sol. Energy 2019, 188, 312–317. 10.1016/j.solener.2019.06.025.

[ref9] Domanski K.; et al. Migration of cations induces reversible performance losses over day/night cycling in perovskite solar cells. Energy Environ. Sci. 2017, 10 (2), 604–613. 10.1039/C6EE03352K.

[ref10] Wenson G.; Thakkar H.; Tsai H.; Stein J.; Singh R.; Nie W. The degradation and recovery behavior of mixed-cation perovskite solar cells in moisture and a gas mixture environment. J. Mater. Chem. A 2022, 10 (25), 13519–13526. 10.1039/D2TA02352K.

[ref11] Huang F.; et al. Fatigue behavior of planar CH3NH3PbI3 perovskite solar cells revealed by light on/off diurnal cycling. Nano Energy 2016, 27, 509–514. 10.1016/j.nanoen.2016.07.033.

[ref12] Khenkin M. V.; et al. Reconsidering figures of merit for performance and stability of perovskite photovoltaics. Energy Environ. Sci. 2018, 11 (4), 739–743. 10.1039/C7EE02956J.

[ref13] Singh R.; et al. Danger in the Dark: Stability of Perovskite Solar Cells with Varied Stoichiometries and Morphologies Stressed at Various Conditions. ACS Appl. Mater. Interfaces 2024, 16 (21), 27450–27462. 10.1021/acsami.4c04350. 38751205

[ref14] Prete M.; et al. Bias-Dependent Dynamics of Degradation and Recovery in Perovskite Solar Cells. ACS Appl. Energy Mater. 2021, 4, 6562–6573. 10.1021/acsaem.1c00588.

[ref15] Lee S.-W.; et al. UV Degradation and Recovery of Perovskite Solar Cells. Sci. Rep. 2016, 6, 38150 10.1038/srep38150. 27909338 PMC5133559

[ref16] Velilla E.; Jaramillo F.; Mora-Seró I. High-throughput analysis of the ideality factor to evaluate the outdoor performance of perovskite solar minimodules. Nat. Energy 2021, 6 (1), 54–62. 10.1038/s41560-020-00747-9.

[ref17] Stoichkov V.; Bristow N.; Troughton J.; De Rossi F.; Watson T. M.; Kettle J. Outdoor performance monitoring of perovskite solar cell mini-modules: Diurnal performance, observance of reversible degradation and variation with climatic performance. Sol. Energy 2018, 170, 549–556. 10.1016/j.solener.2018.05.086.

[ref18] Li J.; et al. Ink Design Enabling Slot-Die Coated Perovskite Solar Cells with > 22% Power Conversion Efficiency, Micro-Modules, and 1 Year of Outdoor Performance Evaluation. Adv. Energy Mater. 2023, 13 (33), 2203898 10.1002/aenm.202203898.

[ref19] Chen W.; et al. Interfacial stabilization for inverted perovskite solar cells with long-term stability. Sci. Bull. 2021, 66 (10), 991–1002. 10.1016/j.scib.2021.02.029. 36654256

[ref20] Ramirez D.; Velilla E.; Montoya J. F.; Jaramillo F. Mitigating scalability issues of perovskite photovoltaic technology through a p-i-n meso-superstructured solar cell architecture. Sol. Energy Mater. Sol. Cells 2019, 195, 191–197. 10.1016/j.solmat.2019.03.014.

[ref21] De Bastiani M.; et al. Toward Stable Monolithic Perovskite/Silicon Tandem Photovoltaics: A Six-Month Outdoor Performance Study in a Hot and Humid Climate. ACS Energy Lett. 2021, 6, 2944–2951. 10.1021/acsenergylett.1c01018.

[ref22] Liu J.; et al. 28.2%-Efficient, Outdoor-Stable Perovskite/Silicon Tandem Solar Cell. Joule 2021, 5 (12), 3169–3186. 10.1016/j.joule.2021.11.003.

[ref23] Khenkin M.; et al. Light cycling as a key to understanding the outdoor behaviour of perovskite solar cells. Energy Environ. Sci. 2024, 17 (2), 602–610. 10.1039/D3EE03508E.

[ref24] Emery Q.; et al. Encapsulation and Outdoor Testing of Perovskite Solar Cells: Comparing Industrially Relevant Process with a Simplified Lab Procedure. ACS Appl. Mater. Interfaces 2022, 14 (4), 5159–5167. 10.1021/acsami.1c14720. 35108814

[ref25] De Rossi F.; et al. An Interlaboratory Study on the Stability of All-Printable Hole Transport Material–Free Perovskite Solar Cells. Energy Technol. 2020, 8 (12), 1–10. 10.1002/ente.202000134.

[ref26] Huang M.; et al. Perovskite Solar Module Outdoor Field Testing and Spectral Irradiance Effects on Power Generation. Phys. Status Solidi - Rapid Res. Lett. 2022, 16 (11), 2–7. 10.1002/pssr.202200220.

[ref27] Gehlhaar R.; Merckx T.; Qiu W.; Aernouts T. Outdoor Measurement and Modeling of Perovskite Module Temperatures. Glob. Challenges 2018, 2 (7), 1800008 10.1002/gch2.201800008. PMC6607244 31565338

[ref28] Pescetelli S.; et al. Integration of two-dimensional materials-based perovskite solar panels into a stand-alone solar farm. Nat. Energy 2022, 7 (7), 597–607. 10.1038/s41560-022-01035-4.

[ref29] Remec M.; et al. From Sunrise to Sunset: Unraveling Metastability in Perovskite Solar Cells by Coupled Outdoor Testing and Energy Yield Modelling. Adv. Energy Mater. 2024, 14, 2304452 10.1002/aenm.202304452.

[ref30] Liu Z.; et al. Machine learning with knowledge constraints for process optimization of open-air perovskite solar cell manufacturing. Joule 2022, 6 (4), 834–849. 10.1016/j.joule.2022.03.003.

[ref31] Al-Sabana O.; Abdellatif S. O. Optoelectronic devices informatics: optimizing DSSC performance using random-forest machine learning algorithm. Optoelectron. Lett. 2022, 18 (3), 148–151. 10.1007/s11801-022-1115-9.

[ref32] Odabaşı Ç.; Yıldırım R. Machine learning analysis on stability of perovskite solar cells. Sol. Energy Mater. Sol. Cells 2020, 205, 110284 10.1016/j.solmat.2019.110284.

[ref33] Lu Y.; et al. Predicting the device performance of the perovskite solar cells from the experimental parameters through machine learning of existing experimental results. J. Energy Chem. 2023, 77, 200–208. 10.1016/j.jechem.2022.10.024.

[ref34] Liu Y.; et al. How Machine Learning Predicts and Explains the Performance of Perovskite Solar Cells. Sol. RRL 2022, 6 (6), 1–11. 10.1002/solr.202101100.

[ref35] Kouroudis I.; et al. Artificial Intelligence-Based, Wavelet-Aided Prediction of Long-Term Outdoor Performance of Perovskite Solar Cells. ACS Energy Lett. 2024, 9 (4), 1581–1586. 10.1021/acsenergylett.4c00328. 38633992 PMC11019640

[ref36] Merckx T.; et al. Stable Device Architecture with Industrially Scalable Processes for Realizing Efficient 784 cm2Monolithic Perovskite Solar Modules. IEEE J. Photovoltaics 2023, 13 (3), 419–421. 10.1109/JPHOTOV.2023.3259061.

[ref37] Kyprianou A.; Giacomin J.; Worden K.; Heidrich M. Differential evolution based identification of automotive hydraulic engine mount model parameters. Proc. Inst. Mech. Eng. Part D J. Automob. Eng. 2000, 214 (3), 249–264. 10.1243/0954407001527402.

[ref38] Herterich J.; et al. Toward Understanding the Short-Circuit Current Loss in Perovskite Solar Cells with 2D Passivation Layers. Sol. RRL 2022, 6 (7), 2200195 10.1002/solr.202200195.

[ref39] Qu G.; et al. Spontaneous decoration of ionic compounds at perovskite interfaces to achieve 23.38% efficiency with 85% fill factor in NiOX-based perovskite solar cells. J. Energy Chem. 2023, 85, 39–48. 10.1016/j.jechem.2023.05.035.

[ref40] Bae S.; et al. Electric-Field-Induced Degradation of Methylammonium Lead Iodide Perovskite Solar Cells. J. Phys. Chem. Lett. 2016, 7 (16), 3091–3096. 10.1021/acs.jpclett.6b01176. 27462013

[ref41] Zuo L.; Li Z.; Chen H. Ion Migration and Accumulation in Halide Perovskite Solar Cells†. Chin. J. Chem. 2023, 41 (7), 861–876. 10.1002/cjoc.202200505.

[ref42] Chen T.; Guestrin C. XGBoost: A scalable tree boosting system. In Proceedings of the ACM SIGKDD International Conference on Knowledge Discovery and Data Mining; 2016; pp 785–794. 10.1145/2939672.2939785.

[ref43] Livera A.; Paphitis G.; Theristis M.; Lopez-Lorente J.; Makrides G.; Georghiou G. Photovoltaic System Health-State Architecture for Data-Driven Failure Detection. Solar 2022, 2 (1), 81–98. 10.3390/solar2010006.

[ref44] Alcañiz A.; Lindfors A. V.; Zeman M.; Ziar H.; Isabella O. Effect of Climate on Photovoltaic Yield Prediction Using Machine Learning Models. Glob. Challenges 2023, 7 (1), 2200166 10.1002/gch2.202200166. PMC9818063 36618102

