# Supplementary material for: Diurnal Changes and Machine Learning Analysis of Perovskite Modules Based on Two Years of Outdoor Monitoring
Source: ACS Energy Lett. 2024 Sep 26;9(10):5081–91. doi: 10.1021/acsenergylett.4c01943 (PMC11605806; doi:10.1021/acsenergylett.4c01943)
Supplement: Supplementary file 1 — nz4c01943_si_001.pdf [file nz4c01943_si_001.pdf]

Supplementary information

Diurnal changes and machine learning analysis of perovskite modules based on two years of outdoor monitoring

Vasiliki Paraskeva[1†,*], Matthew Norton[1,†], Andreas Livera[1,†], Andreas Kyprianou[2], Maria Hadjipanayi[1], Elias Peraticos[1], Aranzazu Aguirre[3,4,5], Santhosh Ramesh[3,4,5], Tamara Merckx[3,4,5], Rita Ebner[6], Tom Aernouts[3,4,5], Anurag Krishna[3,4,5*], George E. Georghiou[1]

[1]PV Technology Laboratory, Department of Electrical and Computer Engineering, University of Cyprus, Nicosia, 1678, Cyprus

[2]PV Technology Laboratory, Department of Mechanical and Manufacturing Engineering, University of Cyprus, Nicosia, 1678, Cyprus

[3]Imec, imo-imomec, Thin Film PV Technology, Thor Park 8320, 3600 Genk, Belgium

[4]Hasselt University, imo-imomec, Martelarenlaan 42, 3500 Hasselt, Belgium

[5]EnergyVille, imo-imomec, Thor Park 8320, 3600 Genk, Belgium

[6]AIT Austrian Institute of Technology, Center for Energy, Giefingasse 2, 1210 Vienna, Austria

*Corresponding authors: vparas01@ucy.ac.cy, anurag.krishna@imec.be

[†]These authors contribute equally to this work.

Supplementary Discussion 1: Mini-modules description

We employed a p-i-n-based device configuration as shown in Figure 1 below. The tested mini-modules consist of different electron transport materials (e.g. LiF/C_{60}/BCP, LiF/C_{60}/LiF). The detailed device description is shown in Table 1.

Table 1: Description of perovskite mini—module devices used outdoors.

**Samples Acronym**	Bottom electrode	Electron Transport Material	Absorber	**Hole Transport Material**	**Top electrodes**	Encapsulation
ETL1_A	ITO	LiF/ C60/ BCP	$FA_{0.8}Cs_{0.2}Pb(I_{0.94}Br_{0.06})_3$	NiO	ITO	2 mm sodalime glass
ETL2_A	ITO	LiF/ C60/LiF	$FA_{0.8}Cs_{0.2}Pb(I_{0.94}Br_{0.06})_3$	NiO	ITO	2 mm sodalime glass
ETL2_B	ITO	LiF/ C60/LiF	$FA_{0.8}Cs_{0.2}Pb(I_{0.94}Br_{0.06})_3$	NiO	ITO	2 mm sodalime glass

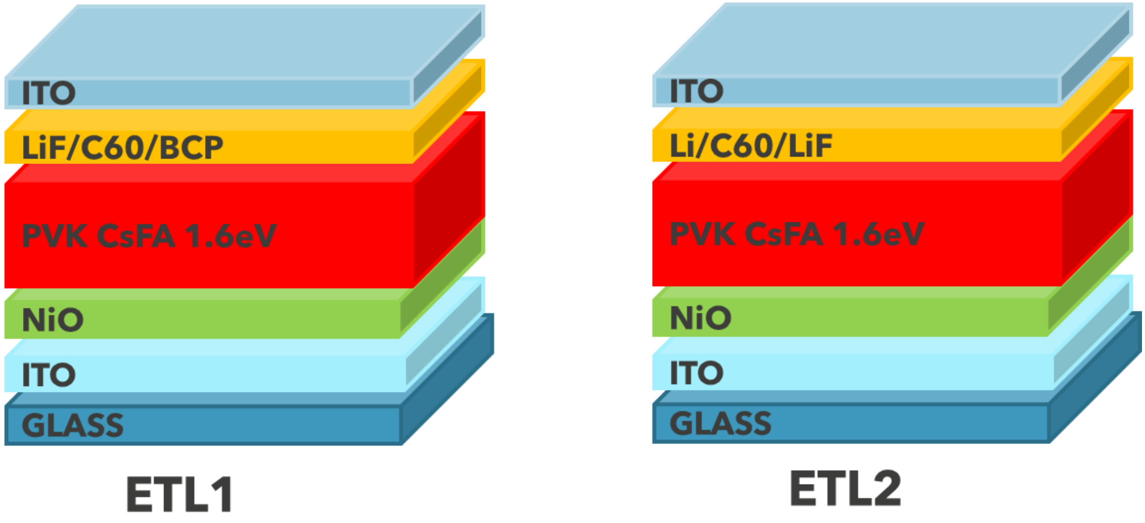

Figure 1: Architecture for the ETL1 and ETL2 devices.

Supplementary Discussion 2: Description of testing operating conditions.

The testing operating conditions (sweep rate, direction of the I-V curve, bias load, start/end voltage, etc.) have been determined for each sample under study. Forward-first approach was selected at almost all instances as this approach was found to minimize hysteresis effects. Reverse-first scan was applied at selected and limited time intervals. Open-circuit load between I-V scans was mainly applied at samples under test apart from some months of testing for some samples. Regarding the sweep rate, the I-V curves were acquired as fast as possible to avoid being affected by any sudden fluctuations in ambient conditions (e.g. irradiance changes due to clouds). For this purpose, a sweep rate of 0.5 V/sec was applied. All the changes at the testing operating conditions are summarized in Table 2. The distribution of the I-V curves per sample is shown in Figure 2.

Table 2: Summary of the outdoor testing operating conditions for each sample under study.

**SAMPLE ACRONYM**	**ETL1_A**	**ETL2_A**	**ETL2_B**
**OUTDOOR EXPOSURE PERIOD**	22/7/2021-	10/8/2022-	10/8/2022-
**BIAS LOAD BETWEEN I-V SCANS**	• Voc (22/7/2021-23/11/2021)<br>• MPP (23/11/2021-4/2/2022)<br>• Voc (4/2/2022-23/5/2022)<br>• MPP (23/5/2022-28/6/2022)<br>• Voc (28/6/2022-)	• Voc (10/8/2022-7/12/2022)<br>• MPP (7/12/2022-7/4/2023)<br>• Voc (7/4/2023-)	Voc
**SCAN DIRECTION PRIORITY**	• Forward-first (22/7/2021-2/9/2022)<br>• Reverse-first (2/9/2021-6/9/2021)<br>• Forward-first (6/9/2021-16/12/2021)<br>• Reverse-first (16/12/2021-4/1/2022)<br>• Forward-first (4/1/2022-)	Forward-first	Forward-first

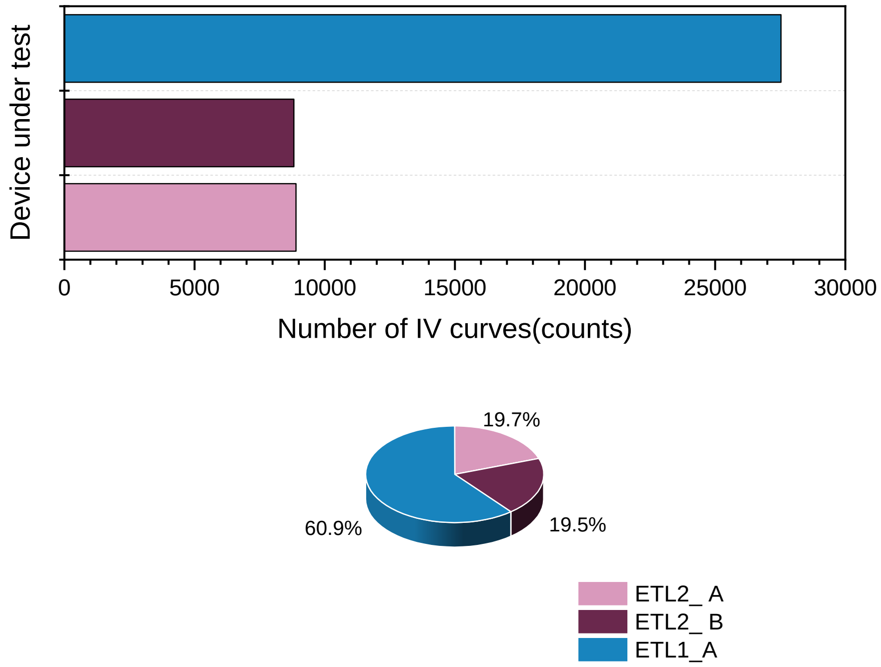

Figure 2:The distribution of the collected I-V curves per sample. The number of I-V curves in the dataset depends on the duration of the outdoor testing.

Supplementary discussion 3: Real-time analysis of the electrical and environmental parameters.

The typical dependence of the modules' characteristics on various environmental parameters during the day is reported. Figure 3 depicts the dependence of open-circuit voltage (V_{oc}), short-circuit current (I_{sc}) and maximum power (P_{max}) measured for the ETL1_A and ETL2_B samples against various environmental parameters, such as solar irradiance (red curve), module temperature (blue curve) and humidity (dark yellow curve) measured for one day. The data of the ETL1 module corresponds to 16th of August 2021 while the data for the ETL2 module corresponds to the 31st of August 2022. Different behavior of the V_{oc} over the day is detected from the two different samples while the behavior of the Isc and Pmax over the day is the same. Humidity levels are getting lower during midday. Real-time analysis of the irradiance, ambient temperature and module temperature for one day in the summer (16/8/2021) can be found in *Figure* 4.

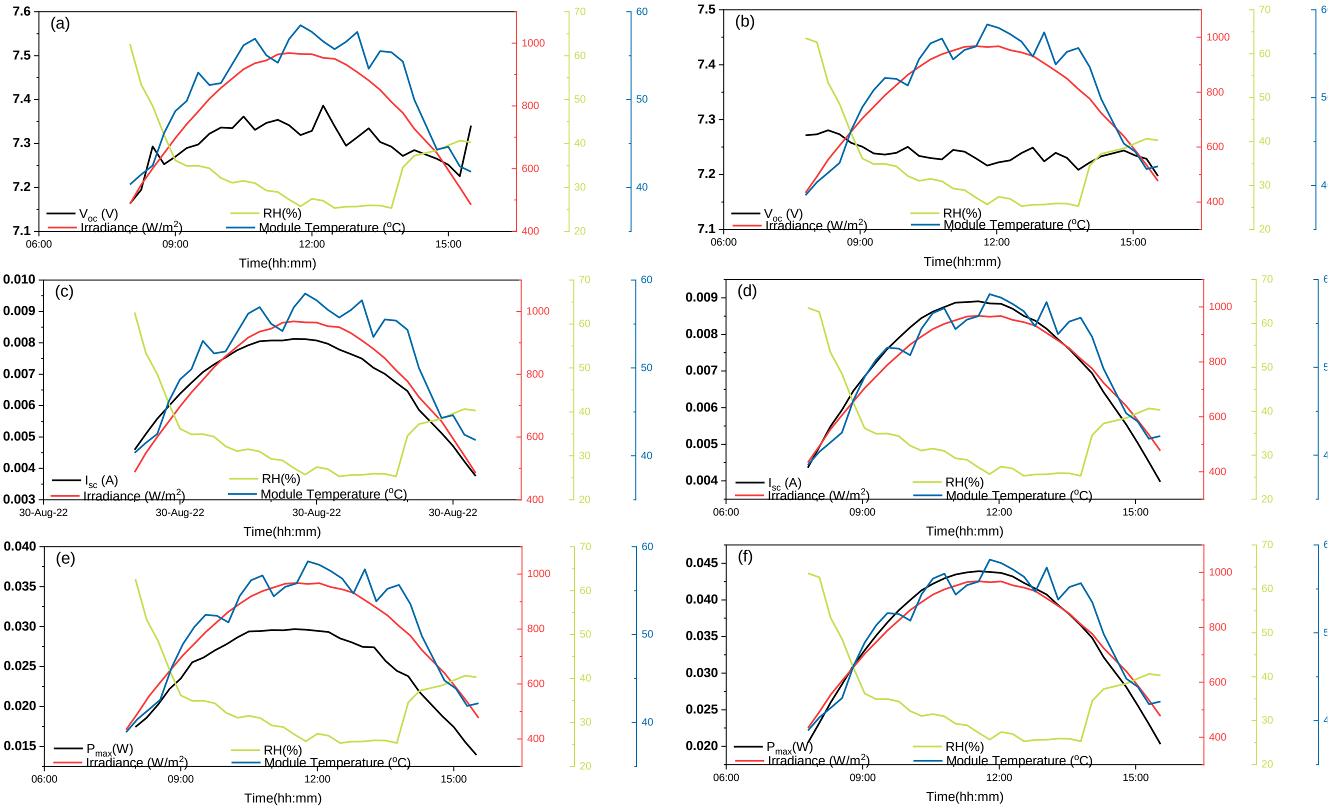

Figure 3: Real-time analysis of the different samples under test. A typical dependence of (a) Voc (c) I_{sc} and (e) P_{max} of the perovskite mini-module ETL1_A with solar irradiance (red curve), module temperature (blue curve), and humidity (yellow curve) during the 16th of August 2021. The dependence of (b) V_{oc}, (d) I_{sc}, and (f) Pmax with irradiance, module temperature, and humidity for sample ETL2_A for the 30th of August 2022 is shown, respectively.

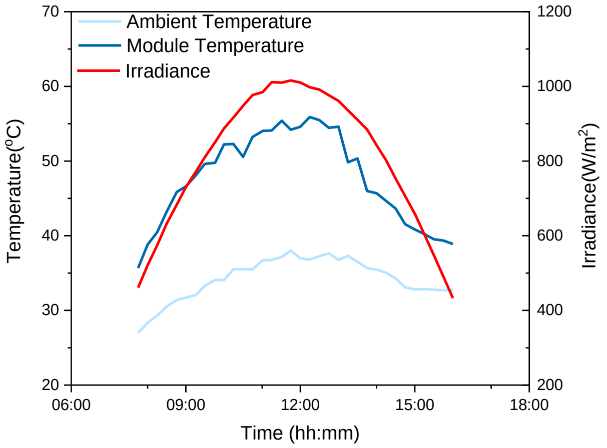

Figure 4: Real-time analysis of the ambient temperature, module temperature and irradiance during the 16th August 2021.

Supplementary discussion 4: Differential evolution algorithm for estimating Performance Loss Rate (PLR)

- Problem Formulation

The daily average performance ratio (PR) of Perovskite modules over a period of a year exhibits a behavior that consists of an initial burn-in period of linear PR loss followed by a period of relatively constant PR. This section describes the fitting of a bilinear model to quantify the behavior described above. The mathematical expression of the bilinear model is given by [1]::

$$PR(Day) = (c_1 + m_1 Day)H + [c_1 + (m_1 - m_2)Day_{tr} + m_2 Day](1 - H) \quad (1)$$
$$H = 1\ if\ Days \leq Day_{tr}$$
$$H = 0\ if\ Days > Day_{tr}$$

where *Day* is the number of days elapsed from the beginning of testing, *PR(Day)* is the performance ratio at *Day* , c_1 the intersection of the initial drop in line with the vertical axis of *PR*, the m_1 is the slope of the initial drop in line, m_2 is the slope of the line corresponding to the second period of almost constant *PR*, Day_{tr} is the day at which the transition from the initial drop in period to the almost constant PR period occurs and *H* is a binary variable taking the value 1 if the days elapsed are less than or equal the transition day Day_{tr} or 0 otherwise. The parameters of model (1), which are mathematically represented as a four-dimensional vector $\mathbf{P} = [c_1\ m_1\ m_2\ Day_{tr}]^T$ that best fit equation 1 to experimental results, are estimated using the experimental measurements of *PR(Days).*

The performance ratio computed using equation (1) for a given vector of parameters, **P**, at a specific day *Day* is denoted by $\widehat{PR}(Days|\mathbf{P})$. The difference between the experimental data and the data obtained using a model with a specific vector of parameters, **P**, over a period of a year is obtained using the mean square error, *MSE,* given by,

$$MSE = \frac{1}{N} \sum_{Days=1}^{N} \left(PR(Days) - \widehat{PR}(Days|\mathbf{P}) \right)^2 \quad (2)$$

where *N* is the total number of days that measurements were taken over a period of a year. Estimation is then mathematically formulated as the following optimization problem:

Find the parameter vector P ̂which minimizes the MSE (2) subjected to the constraint:

$$\mathbf{P}_{min} \leq \widehat{\mathbf{P}} \leq \mathbf{P}_{max} \quad (3)$$

where $\mathbf{P}_{min}$ and $\mathbf{P}_{max}$ are vectors containing the minimum and maximum values of the parameters.

The bilinear PR model, equation (1), contains cross products between the parameters, namely the product between the difference of the slopes and the transition day. In addition, the definition of the bilinear function contains a conditional involving the independent variable DayDay and the parameter DaytrDaytr. This renders the error function non-linear and discontinuous. The discontinuity appears at the point where the conditional is implemented. The consequence of these features of the MSE is that conventional, derivative-based minimization algorithms cannot be used to solve the above optimization problem. In this study,

differential evolution, which is a methodology based on direct search, was chosen to solve the above optimization problem.

Differential evolution (DE) has been developed as an alternative to conventional genetic algorithms to more efficiently handle floating point parameter values in the processes of mutation and crossover. It particularly uses the concept of perturbing a vector of parameters $\mathbf{P}_i$ with the difference o $\Delta\mathbf{P}_i=\mathbf{P}_j-\mathbf{P}_k$ which was adopted from another optimization method, namely that of Nelder and Mead downhill simplex algorithm. The details of the methodology can be found in [2].

The DE algorithm, and in general algorithms that implement direct search optimization, do not prosses a mechanism to restrict the parameters within the range determined by equation (3). To circumvent this, the constrained optimization problem was transformed to an unconstrained one by adding the following penalty function to MSE [3]:

$$Penalty(\mathbf{P}(\mathrm{i})) = \begin{cases} 20\left(\dfrac{\mathbf{P}_{\boldsymbol{min}}(\mathrm{i}) - \mathbf{P}(\mathrm{i})}{\mathbf{P}_{\boldsymbol{min}}(\mathrm{i})}\right)^{\mathbf{2}} & \text{For } \mathbf{P}(\mathrm{i})<\mathbf{P}_{\mathrm{min}}(\mathrm{i}) \\ 0 & \text{For } \mathbf{P}_{\mathrm{min}}(\mathrm{i}) \leq \mathbf{P}(\mathrm{i}) \leq \mathbf{P}_{\mathrm{max}}(\mathrm{i}) \\ 20\left(\dfrac{\mathbf{P}_{\boldsymbol{max}}(\mathrm{i}) - \mathbf{P}(\mathrm{i})}{\mathbf{P}_{\boldsymbol{max}}(\mathrm{i})}\right)^{\mathbf{2}} & \text{For } \mathbf{P}(\mathrm{i})> \mathbf{P}_{\mathrm{max}}(\mathrm{i}) \end{cases} \quad (4)$$

where P(i) is the ith term of the parameter vector P.

The optimization problem that differential evolution solves is the following:

Find the parameter vector $\widehat{\mathbf{P}}$ *which minimizes the cost*:

$$C = MSE + \sum_{i}^{4} Penalty(\mathbf{P}(i)) \quad (5)$$

A brief description of the differential evolution mechanisms of evolving a generation to the next is subsequently given.

- **Differential Evolution Description**

The basic object of the DE algorithm lies in the generation of trial parameter vectors. Its fundamental operations are outlined in Figure 5. In each iteration of differential evolution, a population of vectors evolves into a new population. The table at the top of Figure 5 contains the population of the parameter vectors which will be evolved to the population of the next generation. The parameter vectors of the population of the new generation are listed in the bottom table. The evolution mechanisms of DE are illustrated by the procedure between the two tables.

The first step of the procedure is to select a target vector $\mathbf{P}_t$. It is important to note that all members of the old population are selected as target vectors in turn. Given a target vector, two different vectors from the same population are randomly selected, denoted as $\mathbf{P}_j$ and $\mathbf{P}_k$. Their vectorial difference $\Delta\mathbf{P}=\mathbf{P}_j-\mathbf{P}_k$ is computed and then scaled by a factor F. The scaled difference $F\Delta\mathbf{P}$ is added to a third randomly selected vector $\mathbf{P}_p$ to yield the vector, $\mathbf{P}_m= \mathbf{P}_p+ F\Delta\mathbf{P}$, known as the mutated or perturbed vector. The mutated vector is subsequently cross over with the target vector to generate the trial vector.

The mutated and target vectors are considered the parent vectors, and the vector resulting from the crossover (recombination) operation is considered the child. The child vector Ptr is generated by taking parameters from either parent vector using binomial experimental events.

The likelihood of obtaining the parameters from one or the other parent is determined by the cross-over ratio, CR.

In the version of differential evolution used in this study, the parameters were determined by randomly choosing between two mutually exclusive events: Event A: a certain parameter is taken from Pp with odds CR:1 or Event B: a certain parameter is taken form Pt with odds (1-CR):1. The recombination of the cross over process is depicted in Figure 6.

Finally, the target and trial vector compete for passage to the next generation. The one with the lower cost value advances to the next generation. This procedure repeats for every vector in the population of older generation and, upon its completion, a new generation of parameter vectors with lower costs is generated. The operation of creating newer generations repeats until either a predefined number of generations is created or a parameter vector with a lower cost value that a predefined cost value is generated.

- **Results**

The methodology described above was implemented in two different data sets. The experimental data of performance ratio together with the best bilinear model fitted to these data using the DE methodology are described in the main text. The days when data were not available were not taken into consideration. The bounds of the parameters were set are shown in *Table 3* . The optimal parameter values and MSEs are provided in *Table 4*. A discussion on the interpretation of these values is given in the main text. An additional observation made concerns the values of the minimum MSE.

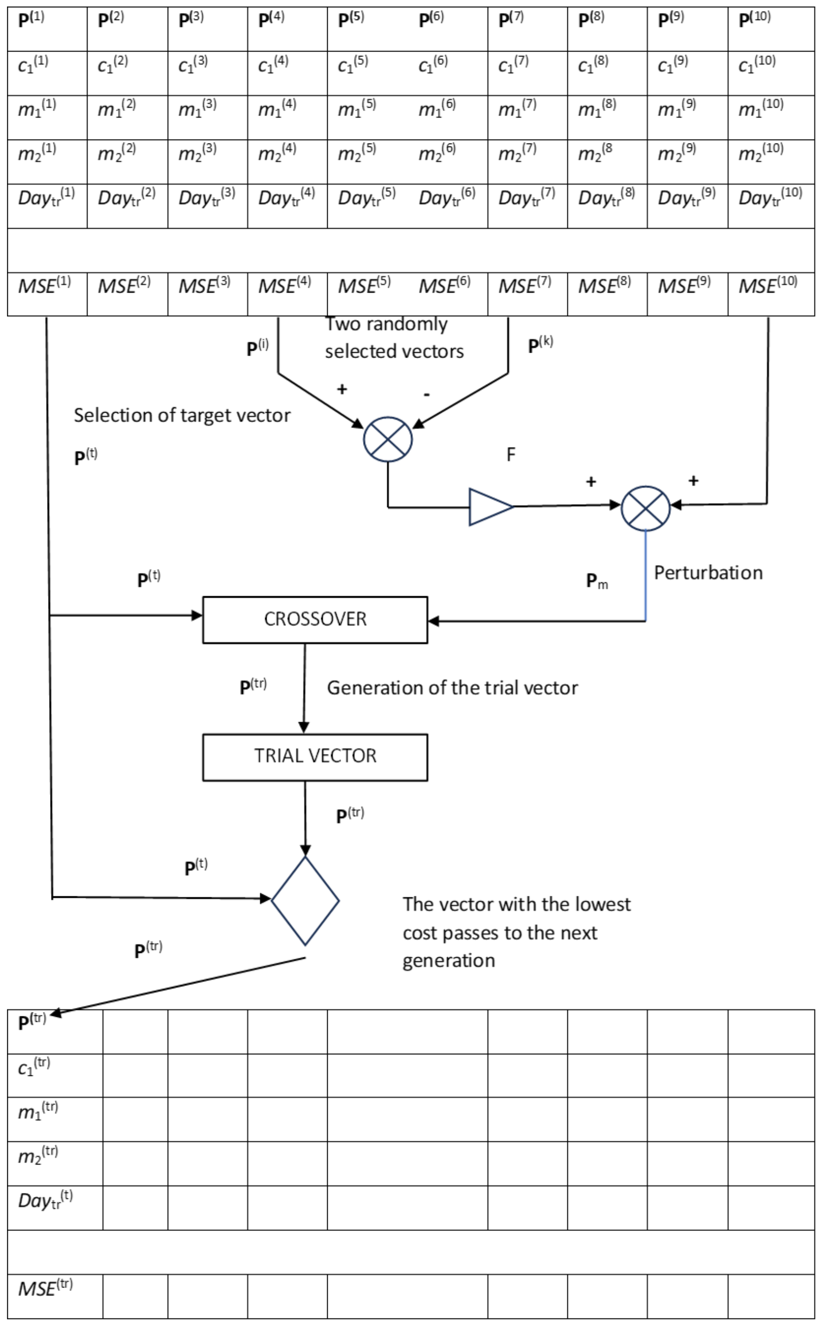

Figure 5: Outline of differential evolution algorithm.

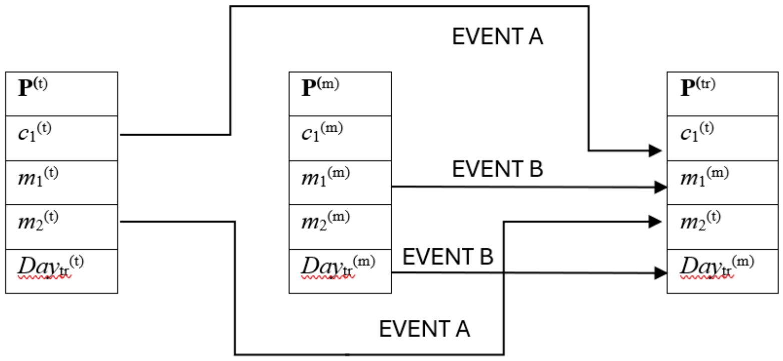

Figure 6: Representation of the cross over process.

Table 3: The upper and lower bounds of the parameters.

	Lower	Upper
c_1	1	100
m_1	-0.3	0
m_2	-0.1	0.3
Day_{tr}	1	365

Table 4: Optimal parameters and MSEs.

	ETL1_A	ETL2_B
c_1	-0.23	-0.21
m_1	78.3	83.4
m_2	-0.013	0.017
Day_{tr}	90.2	91.3
MSE	5.02	14.26

Supplementary discussion 5: Mini-modules performance assessment over time and impact of load on vacancy distribution.

- **Performance assessment of perovskite mini - modules**

The major electrical parameters of the devices under test (P_{max}, I_{sc}, V_{oc}, Fill Factor) were monitored daily in the field for the different perovskite mini-module structures. The evolution

of each parameter over the two years of testing for the ETL1 structure is demonstrated in Figure 7. The evolution of the electrical parameters for the ETL2 series samples can be found in Figure 8. Missing data in the plots correspond to maintenance power cuts occur at the tested site (15/12/2022- 12/1/2023 and 11/5/2023- 25/5/2023). Table 5 gives an example of the different metrics (I_{sc}, V_{oc}, $P_{max,}$ $I_{mp,}$ V_{mp}) collected at each I-V scan. The electrical parameters presented in Table 5 correspond to I-V curves collected from the samples during the second day of outdoor operation and at irradiance close to 900 W/m^2. The IV curves from samples that correspond to the instances of Table 5 are depicted in *Figure* 9. Degradation losses from all the major electrical components of the perovskite mini-modules under test the first month of outdoor testing are summarized in Figure 10.

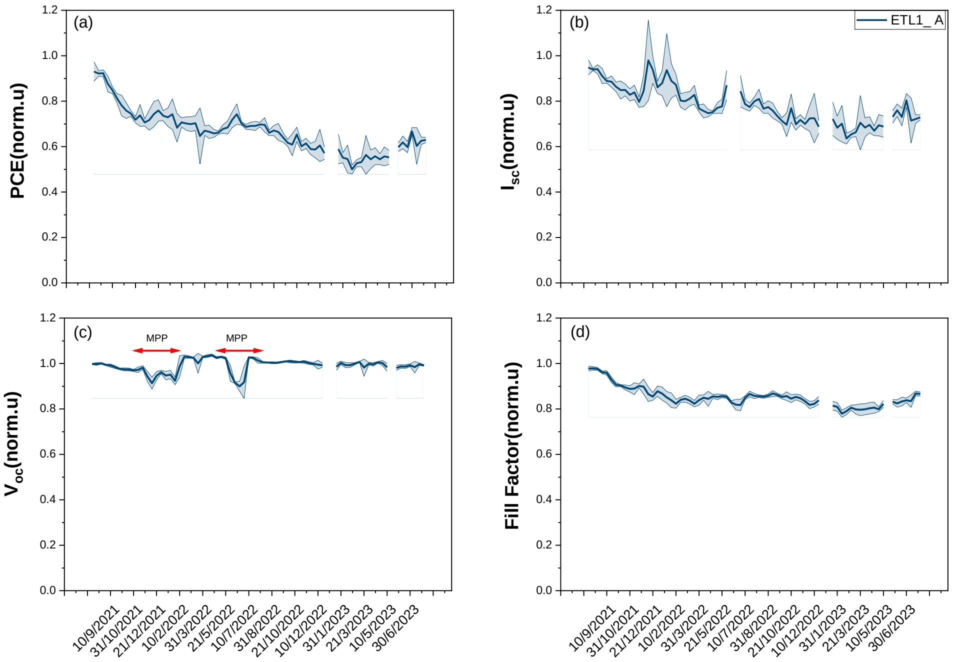

Figure 7: Evolution of (a) PCE, (b) Isc, (c) Voc and (d) Fill Factor for sample ETL1_A over the period of 2 years. Red arrows in (c) indicate the period of MPP application. At all other periods open-circuit voltage between I-V scans was applied.

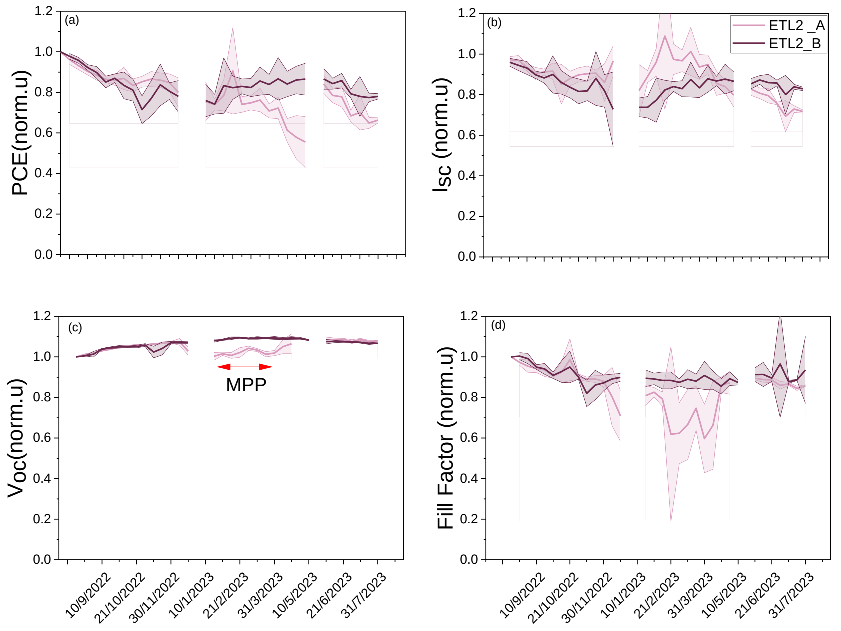

Figure 8: Evolution of (a) PCE, (b) Isc, (c) Voc, and (d) Fill Factor for samples ETL2_ A and ETL2_B over the period of 1 year. The red arrow in (c) indicates the period of MPP application at ETL2_A. At all other periods, open-circuit voltage between I-V scans was applied.

Table 5: Main PV parameters of the I-V curves of the perovskite mini-modules during the second day of outdoor operation and at 900 W/m². The following metrics were collected at each I-V scan: the short-circuit current (I_{sc}), the current at maximum power point (I_{mp}), the voltage at maximum power point (V_{mp}), the power at maximum power point (P_{max}), the open-circuit voltage (V_{oc}), and the Fill Factor (FF).

Mini Module ID	Date & Time	GNI (W/m²)	I_{sc} (mA)	V_{oc} (V)	I_{mp} (mA)	V_{mp}(V)	FF (%)	P_{max} (mW)
**ETL1_A**	23/7/2021 10:25 AM	905	9.67	7.32	7.49	5.32	56.23	39.84
**ETL2_A**	11/8/2022 10:17 AM	896	8.94	7.07	8.04	5.57	70.97	44.85
ETL2_B	11/8/2022 10:20 AM	886	8.89	7.16	7.99	5.68	71.26	45.42

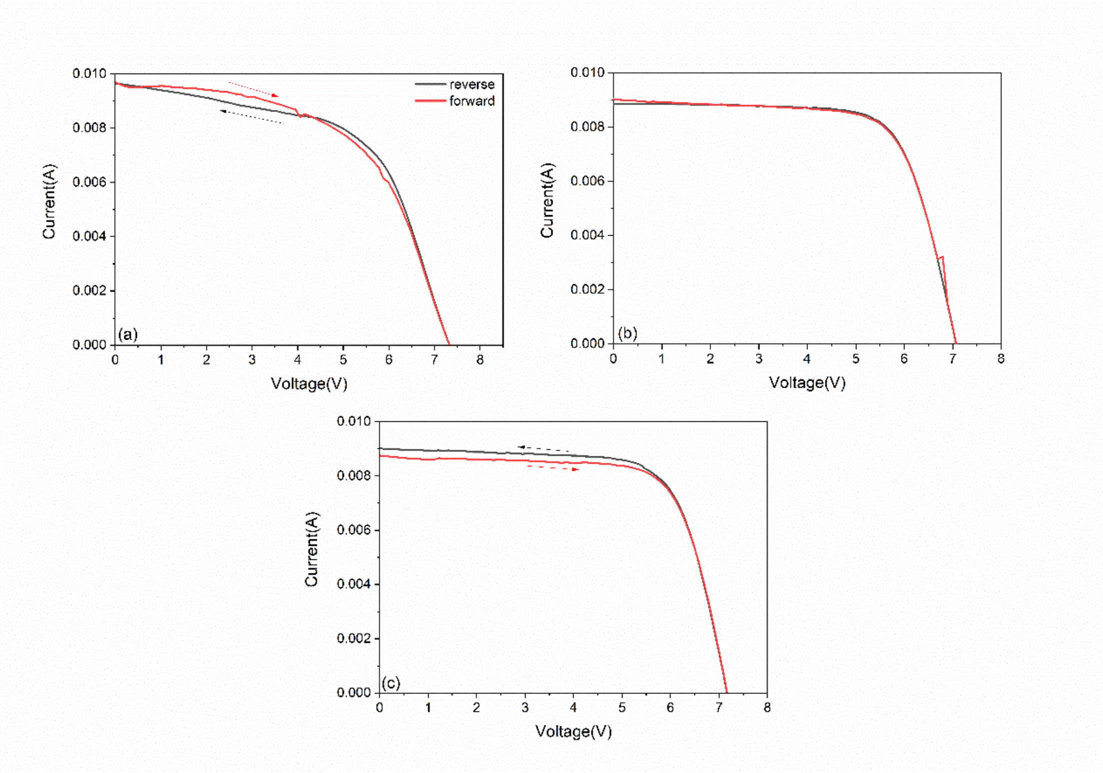

Figure 9: Current-Voltage (IV) curves of samples (a) ETL1_A, (b) ETL2_A and (c) ETL2_B for the instances demonstrated in Table 5.

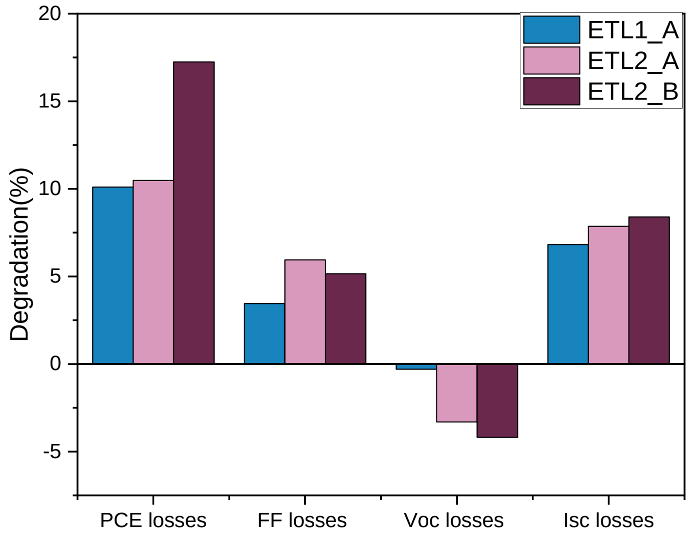

Figure 10: Degradation losses in the first month of outdoor testing from all the major electrical components of the perovskite mini-modules under test.

- **Impact of bias load on vacancy distribution**

In the dark equilibrium state, mobile ions are distributed according to the built-in voltage of the device, which tends to accumulate the ions at the perovskite/transport layer interface. Under illumination, the generated charge carriers counteract the built-in field, reducing the net electric field strength. This reduction in electric field strength decreases ion accumulation at the interfaces and causes ions to diffuse in the opposite direction. The electric field strength is weakest under open circuit conditions. Conversely, at the MPP voltage, the compensation of the built-in field is limited. The effect of ion accumulation at specific interfaces can vary significantly. It may form a potential barrier, increasing the device's series resistance, or expose vacancies that can increase recombination, effectively reducing the open-circuit voltage. It is challenging to determine the nature of the ionic species and their effects solely based on the analysis of IV characteristics, which is a limitation of this study. However, based on the observed behavior, we theorize that under open circuit conditions, the ion distribution is favorable, resulting in fewer vacancies. Conversely, under MPP voltage, more vacancies are exposed. Figure 11 shows the ion distribution in a simulated perovskite device. The pink and blue show the ion and vacancy distribution in the perovskite bulk under 1V and 0.75V respectively.

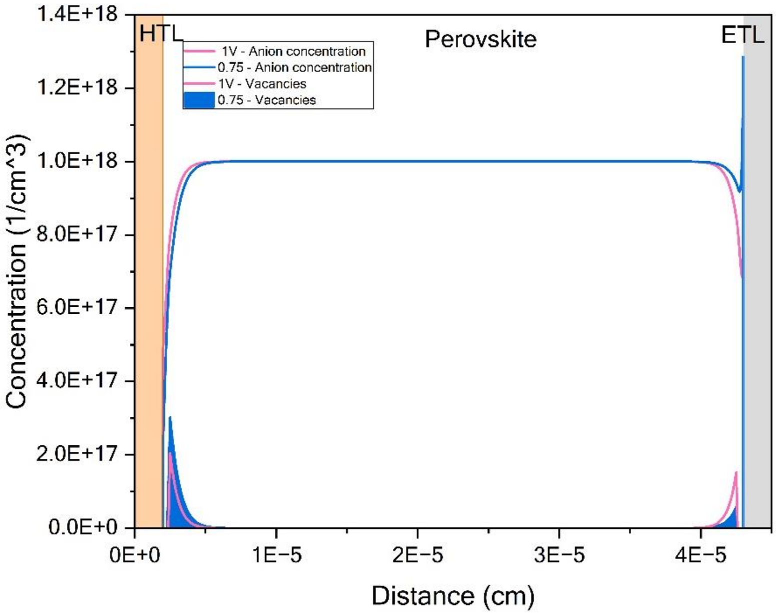

Figure 11: Distribution of ion concentration and vacancies at the HTL/perovskite and ETL/perovskite interface at different bias load conditions.

Supplementary Discussion 6: Diurnal performance degradation and performance recovery overnight calculation.

For the calculation of the DPD and DPR overnight, a dedicated algorithm was built. The algorithm (see Figure 12 and Figure 13) selects the current-voltage characteristics from samples in the morning (at times between 7:00 AM -11:00 AM) and in the afternoon (at times between 15:00 AM – 18:00 AM) at irradiances between 400 W/m^2 and 470 W/m^2. Then filtering for temperature and irradiance levels was performed. Data in the morning and evening that have temperature difference up to 10°C and irradiance difference up to 50 W/m^2 were chosen. The DPD was calculated by selecting the initial and final efficiency value of the

day while DPR overnight was chosen by selecting the last efficiency value of the previous day and the first efficiency value of the next day (see equations 1 and 2 main text, Figure 3 main text and *Figure* 12, *Figure 13* below). The same algorithm was utilized for calculating the diurnal current and voltage degradation and recovery.

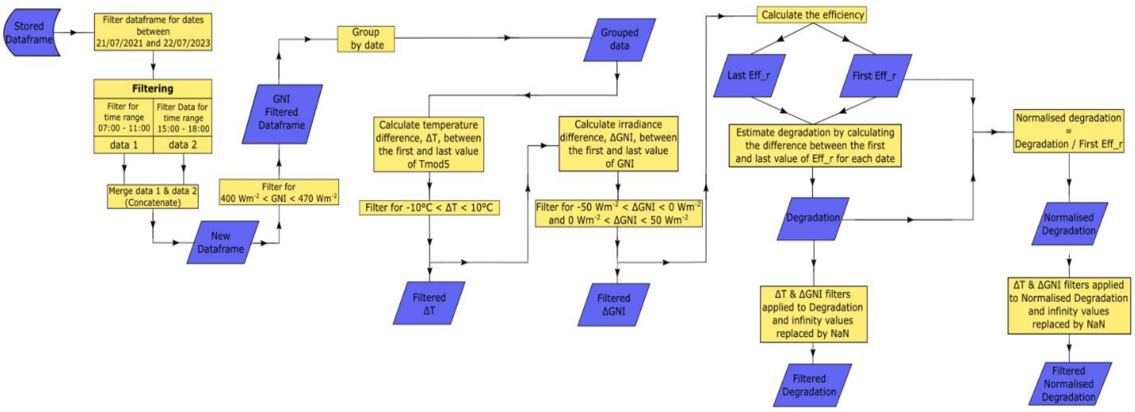

Figure 12: Flow chart of the algorithm utilized to calculate the diurnal performance degradation in perovskite mini-modules under test.

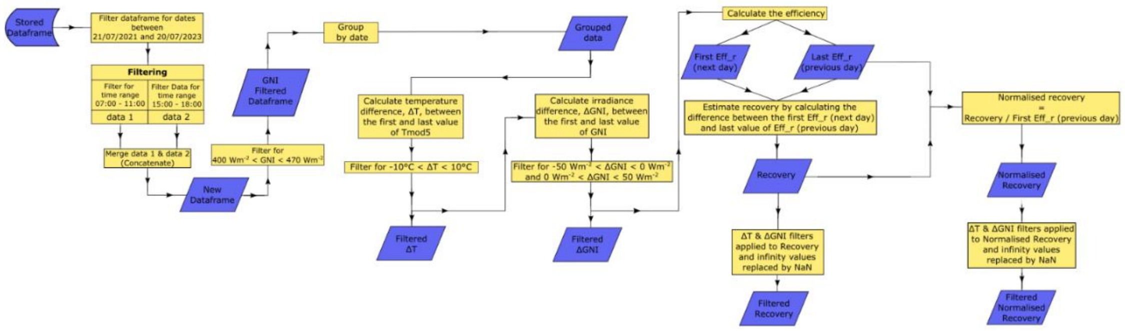

Figure 13: Flow chart of the algorithm utilized to calculate the diurnal performance recovery overnight in perovskite mini-modules under test.

Supplementary Discussion 7: Diurnal Performance Degradation (DPD) and Diurnal Performance Recovery (DPR) of perovskite mini-modules.

The DPD and DPR for sample, ETL2_B were calculated (Figure 14). A sudden increase in DPD and DPR occurred in ETL2_B sample for unknown reason during the period November 2022 and February 2023. The sudden increase of the diurnal changes in that period was found not to be correlated with irradiance or temperature levels since those have been found to be quite stable during the period of the sudden change. Higher statistical error is detected in the winter months where less data has been utilized.

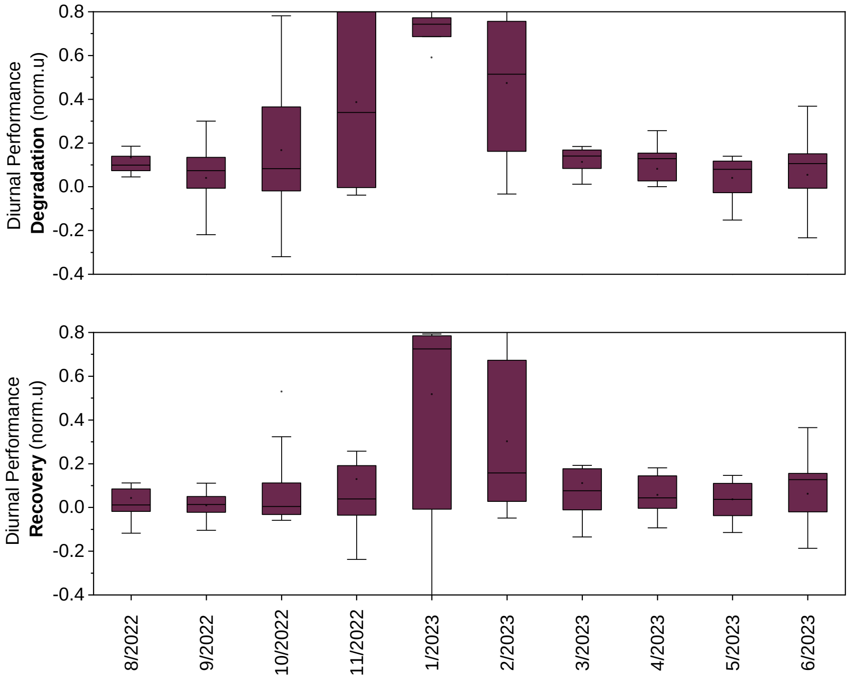

Figure 14: Diurnal performance degradation and diurnal performance recovery overnight for mini-module ETL2_B.

Supplementary Discussion 8: Indoor light-thermal cycling of ETL1 samples

Investigation of stability of two perovskite mini-modules of ETL1 series was implemented under a high throughput aging system in a cycled light-dark analysis and at different temperature levels. Cycled period of 24h and light/dark ratio of 1:1 was utilized. The temperature levels for the one sample are 45°C during light cycles and 23.7°C during dark cycles while for the second sample the temperatures are 23°C during light cycles and 7.5°C during dark cycles. During light cycles, irradiation of 1000 W/m^2 was applied on the samples. The temperature values represent the mean values of temperatures present at the testing site during one typical day in the summer and one typical day in the winter.

The DPD was calculated for each sample based on the equation 1 described in the main text and after 10 hours of light illumination. The results are summarized in Figure 15. A t-test statistical testing was applied on the diurnal values collected from the different temperature cycles and this confirmed that there is a significant difference between the means of both datasets demonstrating the higher diurnal changes in performance at higher temperature levels.

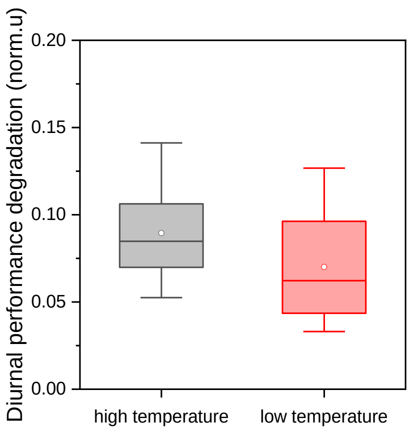

Figure 15: Diurnal performance degradation for two samples exposed at high temperature and low temperature cycles indoors in a light-dark cycling approach. A statistically significant difference was detected between the two different temperature cycles.

Supplementary Discussion 9: Statistical Analysis of diurnal values

Statistical Analysis of DPD and DPR overnight was implemented in all samples under test during the period mentioned in Table 1 (main text). The normalized DPD and DPR overnight were separated into bins of 5% and the frequency of occurrence at each bin was calculated for each module. Figure 16 demonstrates the dominant diurnal degradation and recovery values for the ETL1 and ETL2 samples.

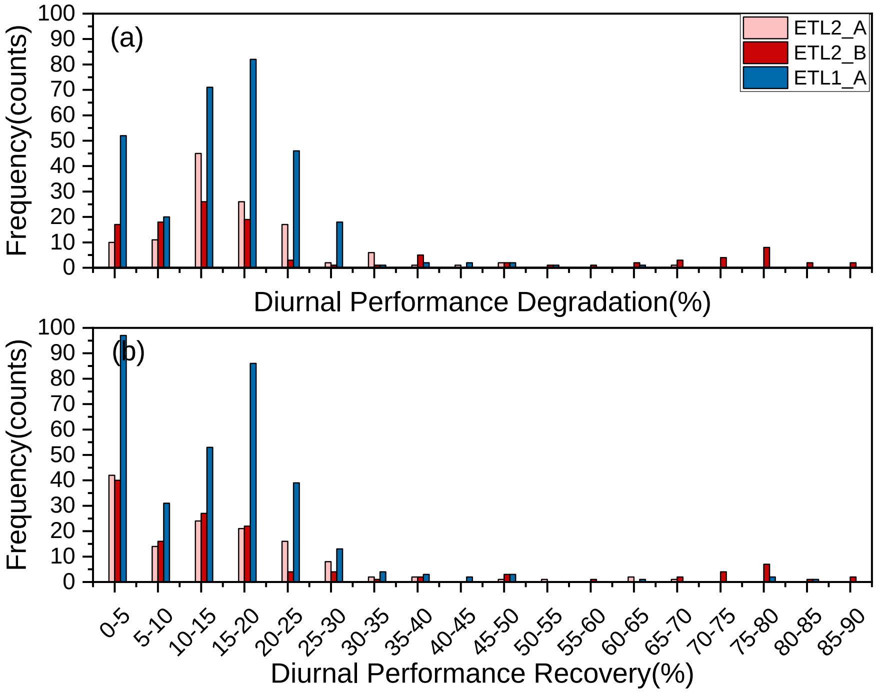

Figure 16: Frequency of occurrence for the (a) Diurnal Performance Degradation and (b) Diurnal Performance Recovery for all perovskite mini-modules under test. Normalized diurnal values were separated into bins of 5%.

Supplementary Discussion 10: Evolution of major electrical parameters over irradiance

The dependence of the maximum power (Pmax), short-circuit current (Isc), open-circuit voltage (Voc) and Fill Factor (FF) on irradiance for all months of testing was studied for the samples of ETL1 series which presented the longest lifetime. Figure 17 below demonstrates the electrical parameters evolution at different irradiance levels in the morning and evening hours during the 14th month of testing of module ETL1_A. This month corresponds to the dates 22/8/2022-22/9/2022. At the specific month of testing, the Voc and FF values were found to have almost identical values in the morning and afternoon while the Pmax and Isc values in the morning and evening present significant differences. The separation of the maximum Pmax and Isc in the morning and evening is different at the different months of testing (see Figure 18). The same holds for Voc and FF values: separation between morning and evening values differs depending on the month of testing. However, in the case of Voc and FF the separation between morning and evening values is much lower compared to that obtained in Isc and Pmax values and in some months (Month 14) is negligible.

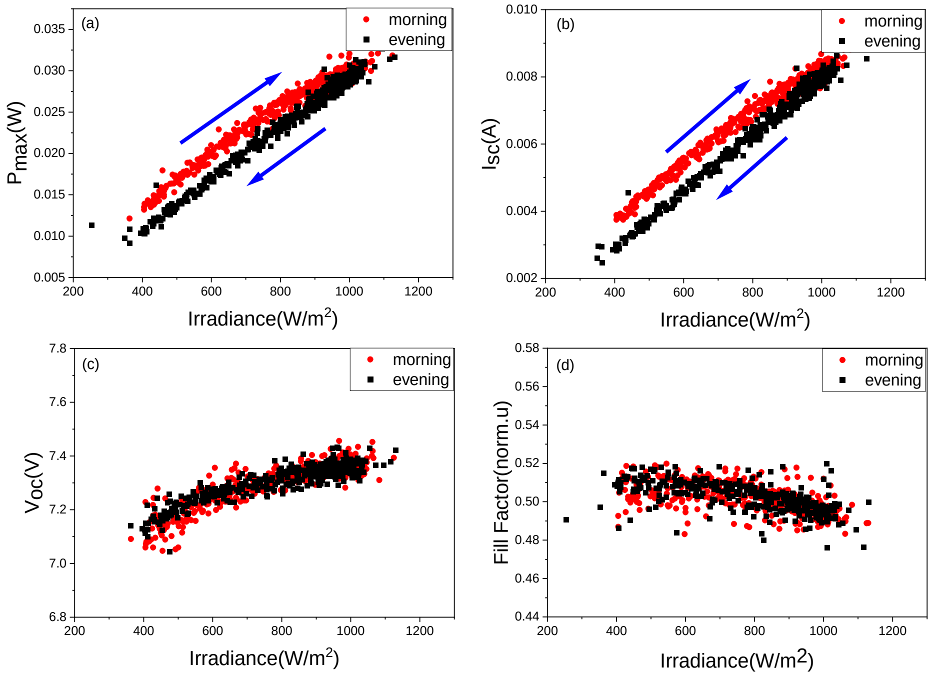

Figure 17: Dependence of mini-module ETL1_A on irradiance in Month 14 (22/8/2022-22/9/2022) (a) Maximum power (P_{max}). (b) Short-circuit current (I_{sc}) (c) Open-circuit voltage (V_{oc}) and (d) Fill Factor (FF).

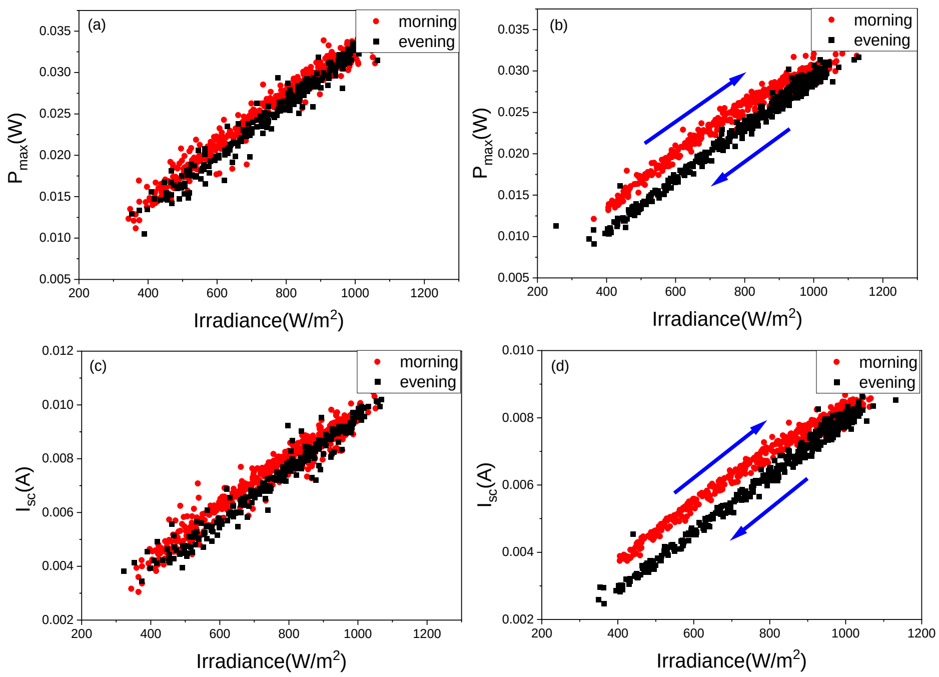

Figure 18: Dependence of P_{max} over irradiance in sample ETL1_A for (a) Month 5 and (b) Month 14. Dependence of I_{sc} over irradiance for (c) Month 5 and (d) Month 14. The Isc and Pmax separation determines the diurnal current and performance degradation values. Month 5 corresponds to dates 22/11/2021-22/12/2021.

Supplementary Discussion 11: Diurnal Degradation and Recovery of maximum power current (I_{mp}), maximum power voltage (V_{mp}) and Fill Factor (FF).

Diurnal degradation and recovery have been calculated for maximum power current (I_{mp}), maximum power voltage (V_{mp}), and Fill Factor (FF). The same algorithm used to calculate the DPD and DPR was utilized in this case (see supplementary Discussion 6). For both type of modules (ETL1 and ETL2) mostly positive values are apparent in diurnal maximum power current (I_{mp}) degradation and recovery (see Figure 19) while mostly negative values are apparent in diurnal maximum power voltage (V_{mp}) degradation and recovery and Fill Factor diurnal values (*Figure 20* and *Figure 21*). Larger statistical error is present at months with small number of data. Low datasets often occur in winter months and in months where power interruption occurred (December 2022, January 2023, May 2023). The difference in FF the morning and evening hours can be found in Figure 22. Diurnal changes occur at all major electrical parameters of the ETL2_B perovskite device during the 16th September 2022 are shown in *Figure* 23. The data corresponds to the 38th day of exposure (where PCE degradation is roughly 8%-10%) and instances for irradiances higher than 400 W/m^2 are shown in the Figure.

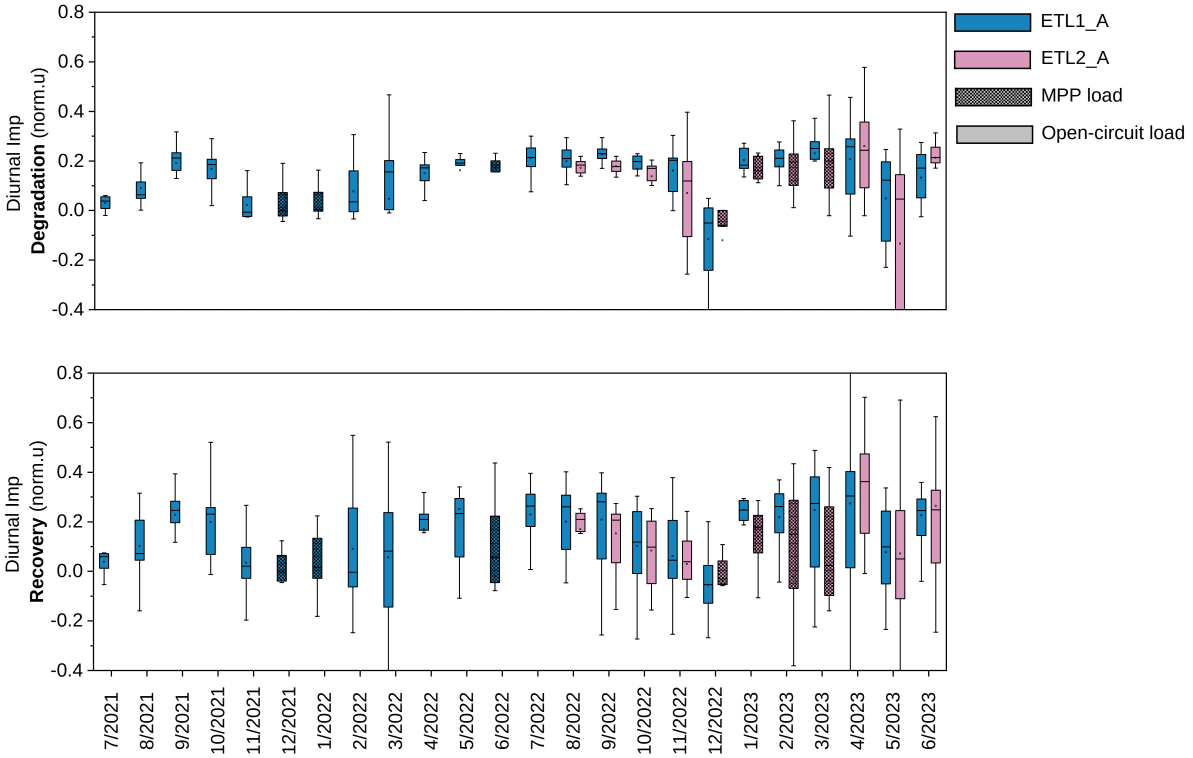

Figure 19: Diurnal Imp (a) degradation and (b) recovery from two represented samples under test.

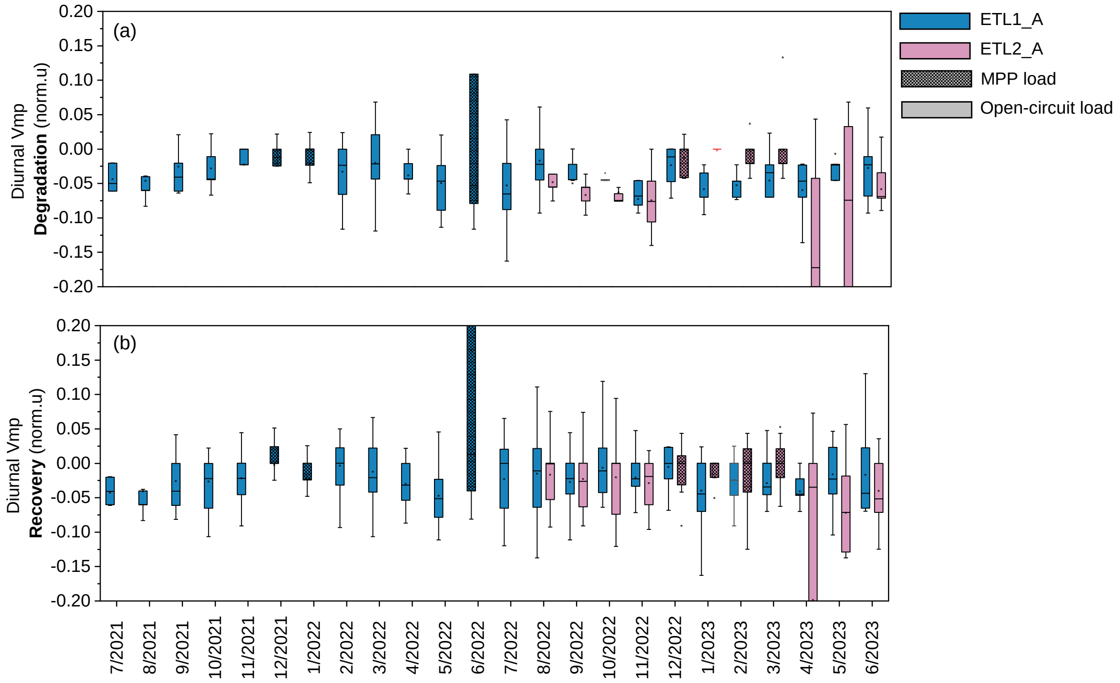

Figure 20: Diurnal Vmp (a) degradation and (b) recovery from two represented samples under test.

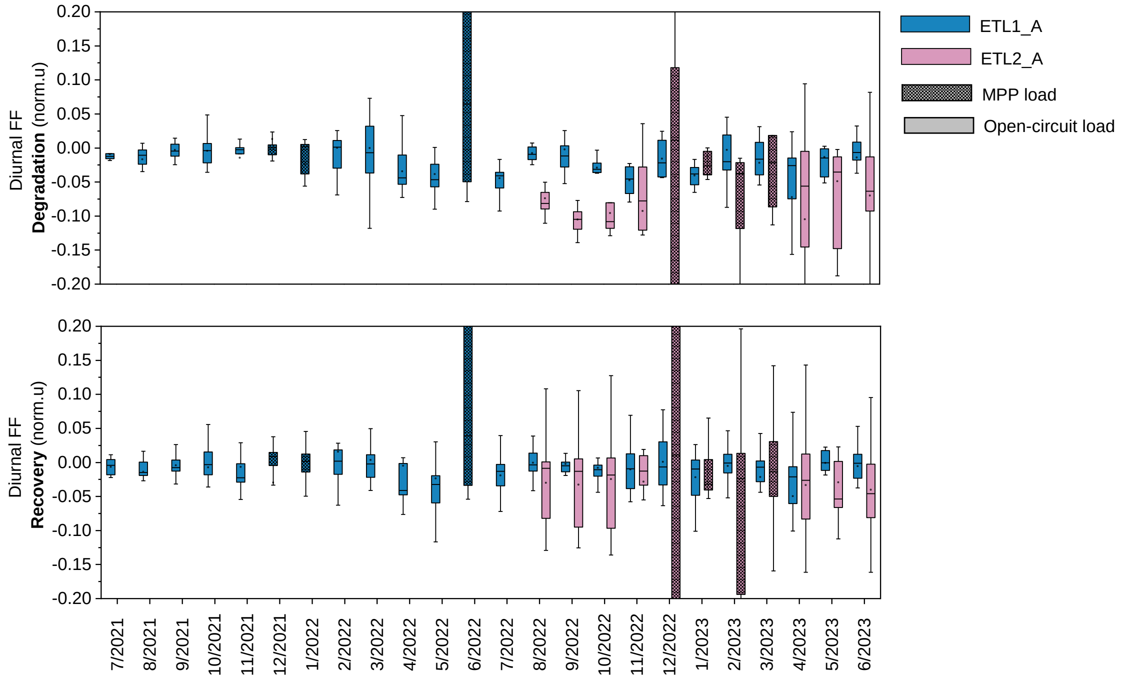

Figure 21: Diurnal Fill Factor (a) Degradation and (b) Recovery from selected samples.

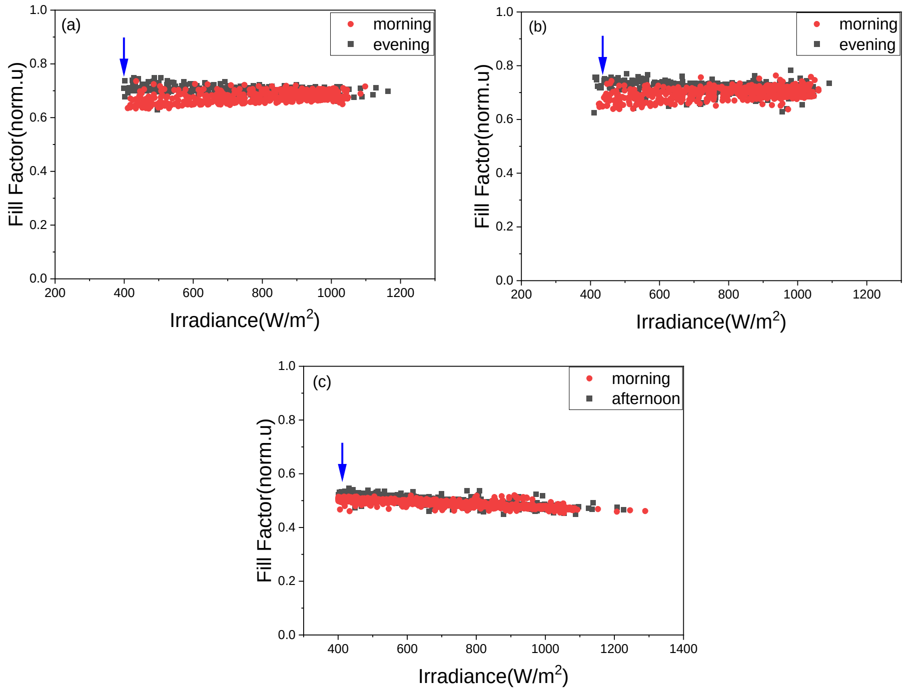

Figure 22: Evolution of Fill Factor over irradiance for samples (a) ETL2_A, (b) ETL2_B, and(c) ETL1_A.. The data from (a) and (b) correspond to Month 1 of testing while the data from (c) correspond to Month 9. The blue arrows at each curve represent the difference in Fill Factor during the morning and evening hours at the same irradiance levels.

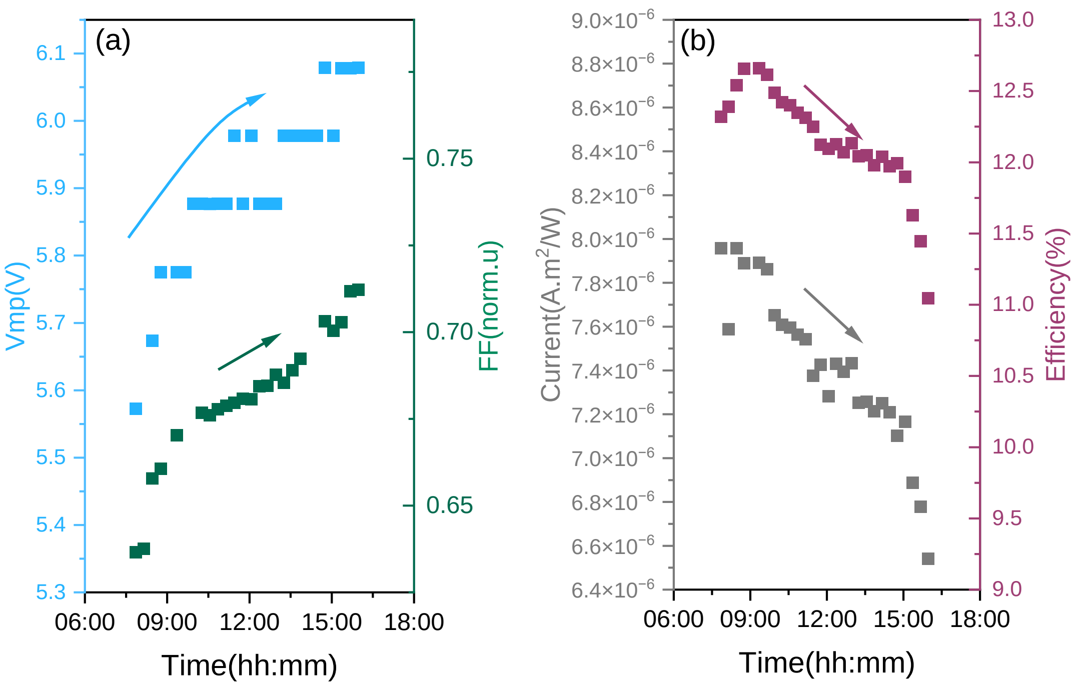

Figure 23: Diurnal changes of (a) Maximum power voltage (Vmp), Fill Factor (FF) and (b)Maximum power current (Imp) and Efficiency for sample ETL2_B during the 16th of September 2022.

Supplementary Discussion 12: Investigation of recovery after storage in the dark

Sample ETL1_A was kept in the dark at ambient conditions (temperature, humidity) for one week (19/6/2023-26/6/2023) after almost two years in the field. The major electrical parameters of the perovskite mini module at irradiance 500W/m^2±25 W/m^2 in the morning hours have been collected before and after the removal of the sample from the outdoor site. Results show short-circuit current and power increasing while open-circuit voltage decreasing. Investigation of the maximum power point current demonstrates an increase after storage in the dark while maximum power voltage exhibits a very slight decrease. All the results are summarized in Figure 24.

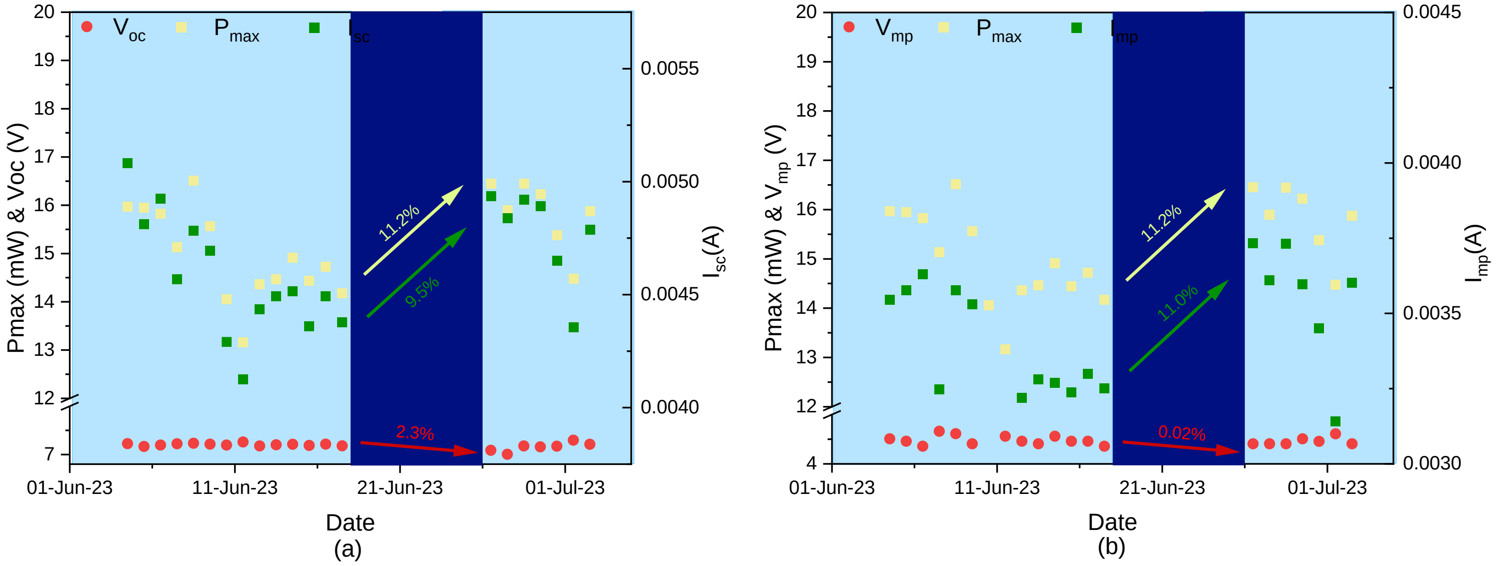

Figure 24: (a) Maximum power (Pmax), short--circuit current (Isc) and open circuit voltage (Voc) of sample ETL1_A at irradiance level 500 W/m² ±25 W/m² from selected dates in June-July 2023.(b) Maximum power (Pmax), maximum power current (Imp) and maximum power voltage (Vmp) of the same module at irradiance level 500 W/m² ±25 W/m² at the same dates. The sample was located indoors in the dark between 19th June 2023 and 26th June 2023. Change of the values of the electrical parameters after storage in the dark are indicated in the graphs.

Supplementary Discussion 13: Analysis of Diurnal Performance Degradation-to-Recovery ratio

Diurnal performance degradation (DPD) and diurnal performance recovery overnight (DPR) were calculated for each day in the field for the modules that presented lifetime up to a year. The absolute values of DPD and DPR were calculated for each day in the field using the following respective equations:

$$Diurnal\ Performance\ Degradation\ (absolute\ value) = PCE_{MORNING\ \left(GNI=400\frac{W}{m^2}\right)} - PCE_{EVENING\left(GNI=400\frac{W}{m^2}\right)}$$

$$Diurnal\ Performance\ Recovery\ (absolute\ value) = PCE_{MORNING,NEXT\ DAY\ \left(GNI=400\frac{W}{m^2}\right)} - PCE_{EVENING,PREVIOUS\ DAY\left(GNI=400\frac{W}{m^2}\right)}$$

The absolute DPR against absolute DPD was plotted for ETL1 and ETL2 series samples (Figure 25 and Figure 26). Larger value of the recovery-to-degradation slope indicates less performance degradation of the perovskite mini-module under test.

The diurnal I_{mp} degradation against I_{mp} recovery was found to be linear while the linearity between degradation and recovery does not hold for the remaining parameters (V_{mp}, FF). The results are summarized in Figure 27.

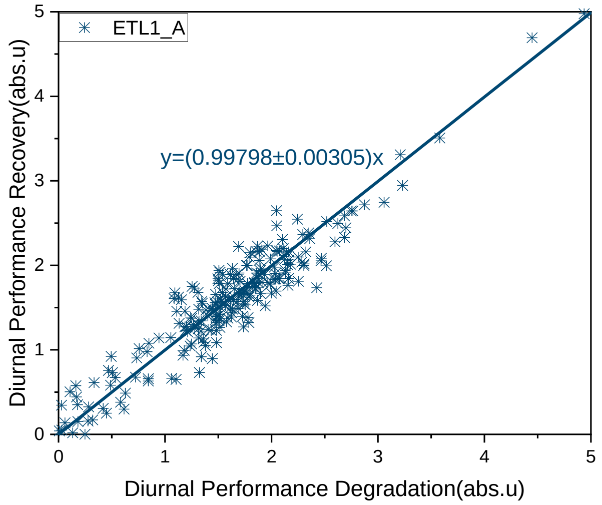

Figure 25:Diurnal performance recovery overnight against diurnal performance degradation for the one sample of ETL1 series. The value of the slope determines the amount of degradation in the perovskite material.

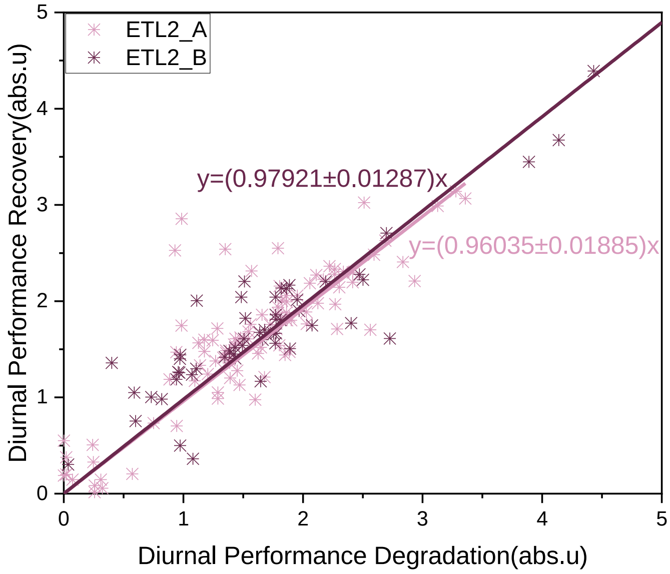

Figure 26:Diurnal performance recovery overnight against diurnal performance degradation for the two samples of ETL2 series.

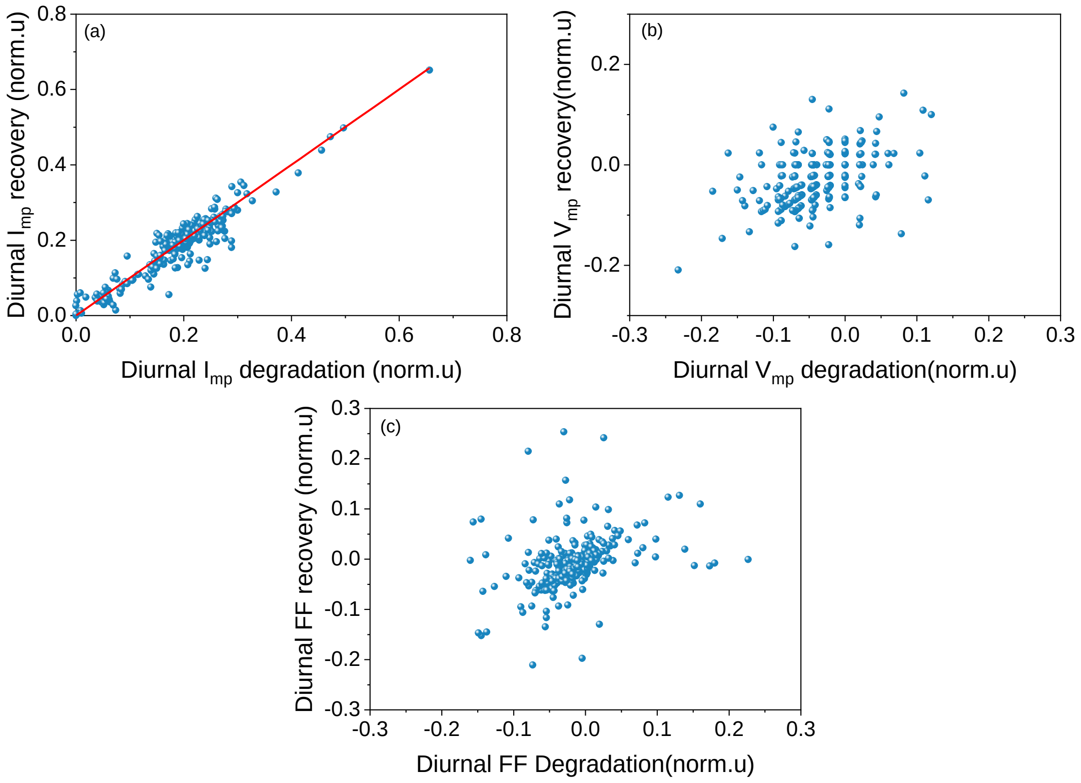

Figure 27: Diurnal recovery against degradation for (a) Maximum power point current Imp (b) Maximum Power Point Voltage Vmp and (c) Fill Factor. The results correspond to ETL1_A that tested outdoors for two years.

Supplementary Discussion 14: Machine learning analysis of two-year dataset from perovskite mini-module ETL1_A

- **Perovskite system Machine Learning Model**

A data-driven predictive model was constructed to simulate the PV performance using the eXtreme Gradient Boosting (XGBoost) regression model. The model was constructed using different model construction conditions (i.e., input features, partitions of actual and synthetic generated performance data for the training procedure, etc.) to simulate the PV performance. The ultimate scope of this procedure is to develop an accurate predictive model that would utilize low shares of data and commonly monitored input features. In case of measured data unavailability, a synthetic generated PV dataset (using measured meteorological data from the test PV installation) was utilized for the development of the predictive model. The model's accuracy is finally evaluated using a yearly PV dataset, containing 10-15-minute field measurements obtained from the perovskite mini-modules installed in Nicosia, Cyprus. Data from the module ETL1_A with the larger lifetime were considered. The actual electrical and weather data acquired for the perovskite mini-modules in the field collected every 10 – 15 minutes over a yearly evaluation period (from July 2021 to June 2022) were used to create the actual performance time series. The constructed time series was used for the predictive model development.

The core of XGBoost Regression model is to optimize the objective function's value by using gradient descent to create new trees based on the residual errors of previously trees. For a given dataset with *n* labelled examples and *m* features, *K* additive functions are used to predict the class of the examples:

$$\hat{y} = \varphi(X_i) = \sum_{k=1}^{K} f_k(X_i),\ f_k \in F \quad (1)$$

where $F = \{f(x) = w_{q(x)}\}(q: \Re \rightarrow T, w \in \Re^T)$ is the space of regression trees, *q* represents the structure of each terminal node index and *T* is the number of leaves in the constructed tree. Each f_k corresponds to an independent tree structure (*q*) and leaf weights (*w)*. To this end, XGBoost minimizes the following regularized objective:

$$L = \sum_i l(\hat{y}_i, y_i) + \sum_k \Omega(f_k) \quad (2)$$

where $\Omega(f) = \gamma T + \frac{1}{2}\lambda\|w\|^2$, *l* is the loss function of the model based on the training data, and *Ω* the regularization term which penalize the complexity of the model. To speed up the optimization of the model second order approximation is used:

$$L^{(t)} \approx \sum_{i=1}^{n}[l(y_i, \hat{y}_i^{(t-1)} + g_i f_t(x_i) + \frac{1}{2}h_i f_t(x_i)] + \Omega(f_t) \quad (3)$$

where $g_i = \partial_{\hat{y}_i^{(t-1)}} l(y_i, \hat{y}_i^{(t-1)})\ and\ h_i = \partial^2_{\hat{y}_i^{(t-1)}} l(y_i, \hat{y}_i^{(t-1)})$ is the first and second order gradient statistics on the loss function, respectively.

The predictive over actual power at different irradiance and temperature levels for the perovskite mini-module under study can be found in Figure 28.

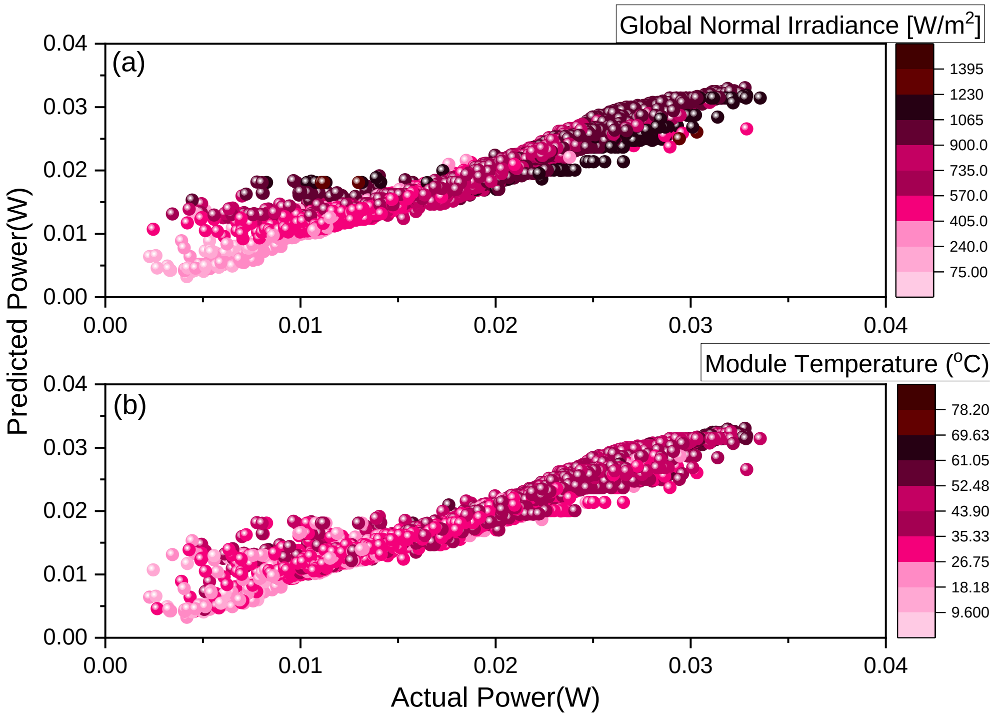

Figure 28: Predicted vs. actual power for perovskite mini-module ETL1_A at different (a) global normal irradiance conditions and (b) module temperatures.

- **Performance metrics**

To evaluate the predictive accuracy of the constructed model, the performance metrics of the root mean squared error (RMSE) and the normalized root mean square error (nRMSE) metrics were used [4]. The RMSE was calculated as follows [4]:

$$RMSE = \sqrt{\frac{1}{N}\sum_{i=1}^{N}(e_i)^2} \quad (4)$$

where N is the number of predictions and e_i is the error between the observed (y_i) and the predicted value $(\hat{y}_i)$ given by:

$$e_i = y_i - \hat{y}_i \quad (5)$$

Likewise, the nRMSE is calculated as follows:

$$nRMSE = \frac{RMSE}{mean(y_i)} \quad (6)$$

Another performance metric utilized in this work was the mean bias error (MBE) and the normalized mean bias error (nMBE). The MBE was calculated as follows:

$$MBE = \frac{1}{N}\sum_{i=1}^{N} e_i \quad (7)$$

where N is the number of predictions and e_i is the error between the observed (y_i) and the predicted value $(\hat{y}_i)$ as provided in equation 5.

The nMBE is calculated as follows:

$$nMBE = \frac{MBE}{\frac{\sum_{i=1}^{N} y_i}{N}}$$

(8)

The nRMSE parameter was calculated at different power levels of the perovskite mini-module under investigation (see main text) and also for each day of the test set period (Figure 29). *Figure 29* depicts that nRMSE of roughly 5.97% was found over the first 100 days of the test set period.

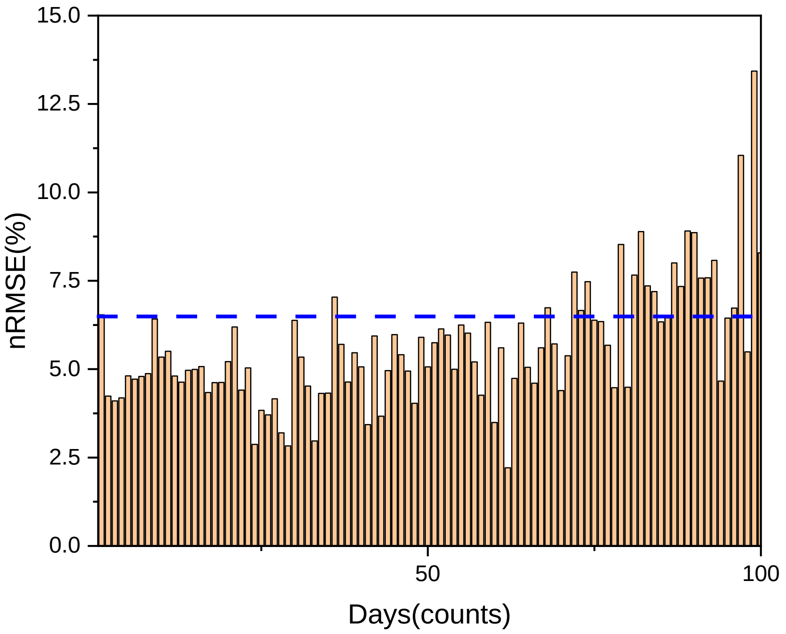

Figure 29: Prediction performance of the XGBoost model given by the daily nRMSE over the first 100 days of the test set period.

Supplementary Discussion 15: Laser scribing for fabrication of perovskite mini-modules

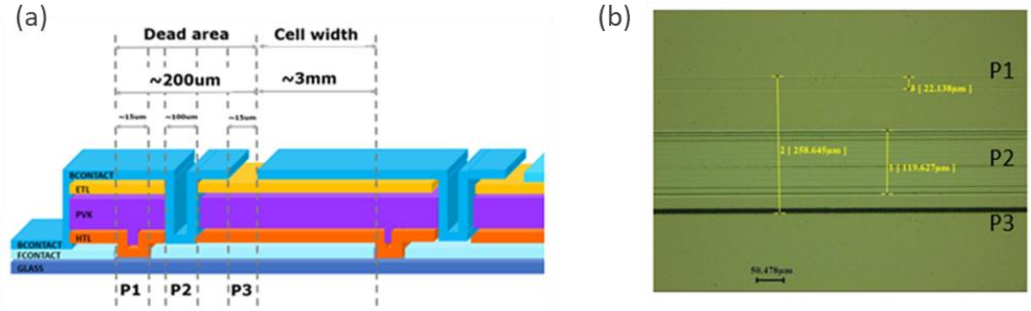

Figure 30: I-V output (a) Illustration of the dead area compared to the active area per cell (image is not in scale), (b) Confocal microscopy image of P1P2P3 scribes.

The module cells are monolithically interconnected in series by using a ps laser (AOWave). The P1 and P3 scribes isolate the cells from each other and the P2 scribe connects the front of one cell to the back of the next one (Figure 30a). P1 scribes remove the front ITO and divide the active area into the desired number of cells (for 4cm2: 7 cells). P1 scribes were performed with an IR ps pulsed laser working at a fluence of 1.70 J/cm². The width of the P1 lines is about 20µm. P2 lines, removing all layers except for ITO, are scribed at a fluence of 0.001 J/cm². The width of the P2 scribe is about 100µm to guarantee optimal electrical contact between the front and back electrodes. Finally, P3 scribes, ablating the back contact layer were marked at a distance from P1 at a fluence of 280 mJ/cm². The width of the P3 scribe is about 15µm. An illustration of the layer stack and the monolithic interconnection scribes appears in Figure 30b). The distance between P1 and P3 scribes, about 258um (Figure 30 a) determines the dead area of the module and leads to a geometrical fill factor of around 91%. After this step, the modules were characterized (initial IV curves) and laminated. The Sn-coated Cu busbars, which were 50 µm thick, were attached using conductive glue (Nagase Chemicals, DB-1588-4). The lamination package included a polyolefin encapsulant (Borealis, BPO8828F) and two 2 mm-thick soda lime glass plates, serving as the front and back sheets. The edges were sealed using butyl rubber (Quanex, Solargain LP-03). The lamination was performed at 130°C for 15 minutes at 1 atm pressure over the sample using an P. Energy Lab laminator (L036LAB). IV curves of the laminated modules were collected prior to shipment to the university of Cyprus. In Figure 31 an image showcasing the laminated assembly is presented.

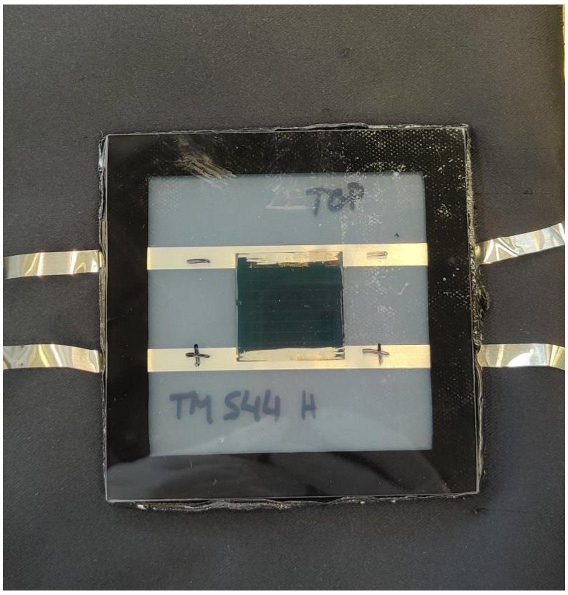

Figure 31: Image of a laminated minimodule.

Supplementary Discussion 16: Outdoor monitoring description

The performance assessment of the perovskite mini-modules was implemented at the FOSS Research Centre for Sustainable Energy which is located at the University of Cyprus campus, Nicosia, Cyprus (latitude: **35° 11' 8.0376'' N**, longitude: **33° 22' 56.1900'' E**). The collection of various electrical (current, voltage, power) and environmental parameters (wind speed, ambient temperature, irradiance at the plane of array, humidity, etc.) is controlled by a Delphin TopMessage Data Acquisition system. A relay control board is connected to a multiplexer and then to 14 relay channels. A Keithley 2430 source-meter collects the current-voltage characteristics of the samples sequentially with a **measurement frequency of roughly 10- 15 minutes**. The samples were located on a solar tracker at a fixed-plane array. Details about the infrastructure can be found in Figure 32.

(a)

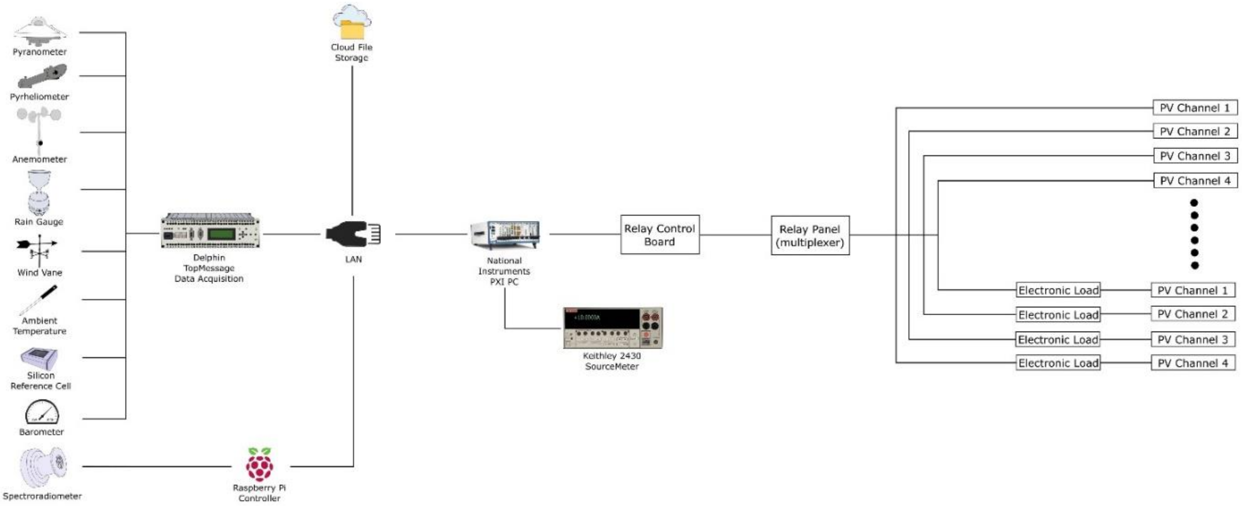

(b)

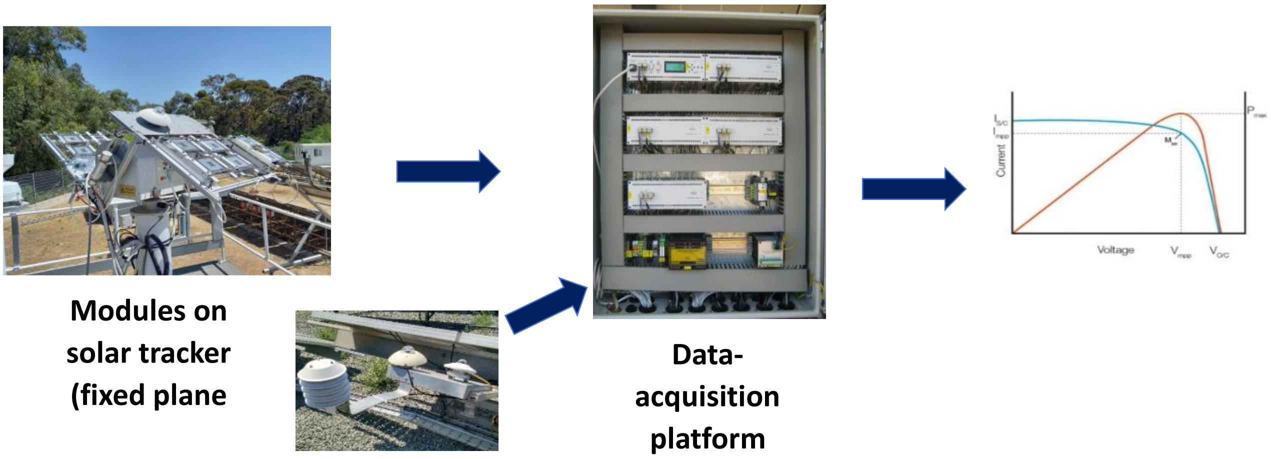

Figure 32: (a) Schematic of the FOSS Lab facilities architecture and (b) Block diagram of the outdoor testing set-up utilized in this work.

Supplementary Discussion 17: Weather Data in the Installation Field

Details regarding the temperature and precipitation levels at the tested location are provided in Figure 33. The map of global irradiation potential of Cyprus is included in Figure 33. A dedicated weather station with a data acquisition system were utilized to monitor the environmental data such as wind speed and direction, sun irradiation, humidity, and temperature (both ambient and module). Statistical analysis of the major weather variables at the tested location is provided in Figure 35. Irradiation (kWh/m^2) in plane of array in the installation field from July 2021 until July 2023 was also calculated based on the sun irradiation data (see Figure 34).

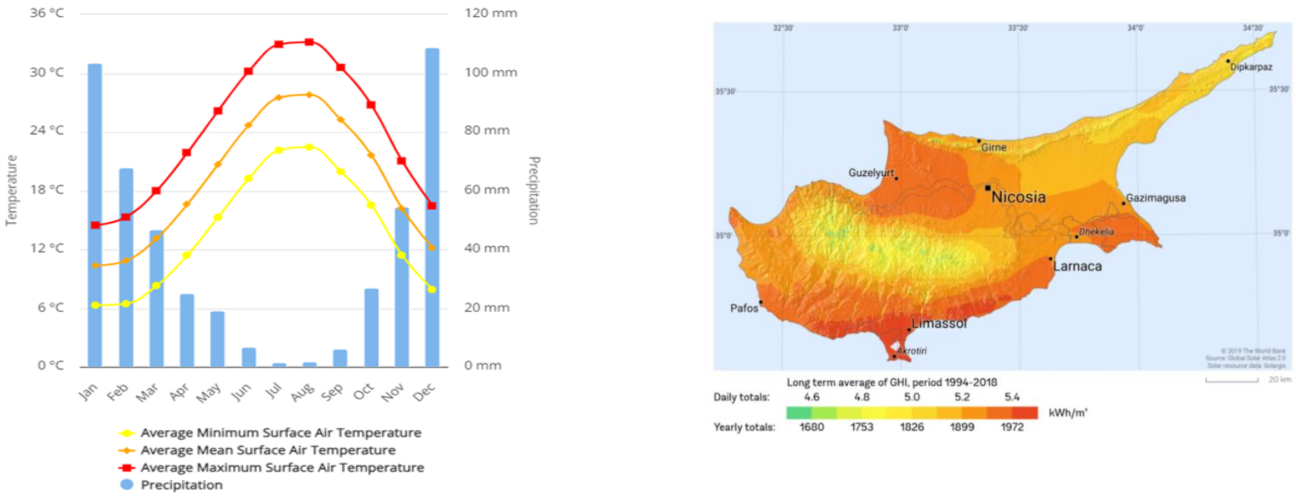

Figure 33: (left) Monthly climatology of average minimum surface air temperature, average mean surface air temperature, average maximum surface air temperature and Precipitation (source: Cyprus - Climatology | Climate Change Knowledge Portal (worldbank.org)) (right) Cyprus global horizontal irradiation map.

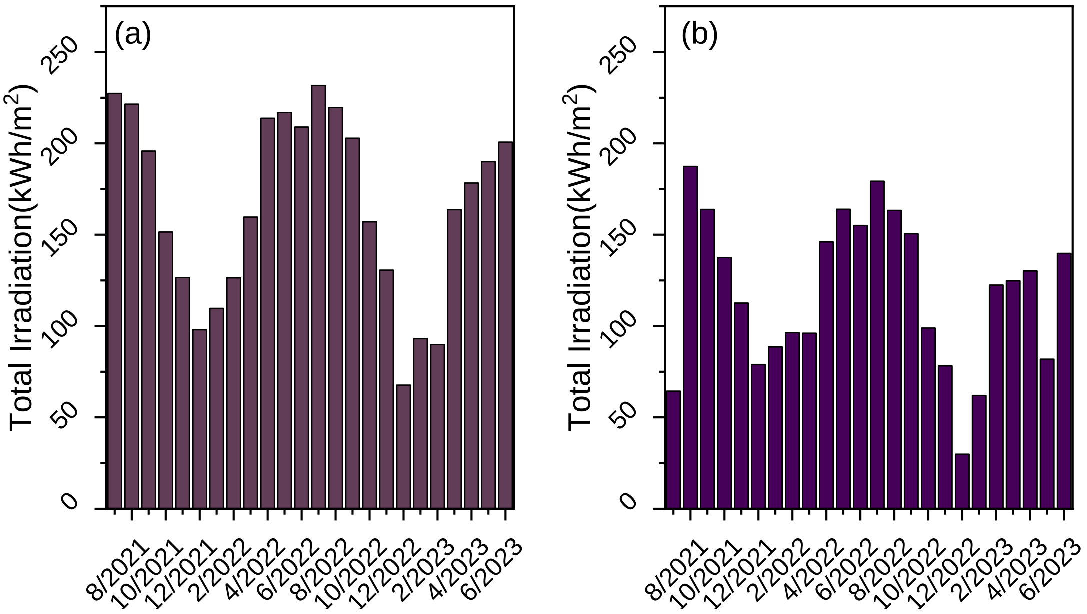

Figure 34: (a) Monthly irradiation at FOSS testing site in plane of 32° during the two years of outdoor exposure of samples (July 2021-July 2023). (b) Monthly irradiation applied on the mini-modules at irradiances above 400W/m² where the diurnal performance degradation was calculated.

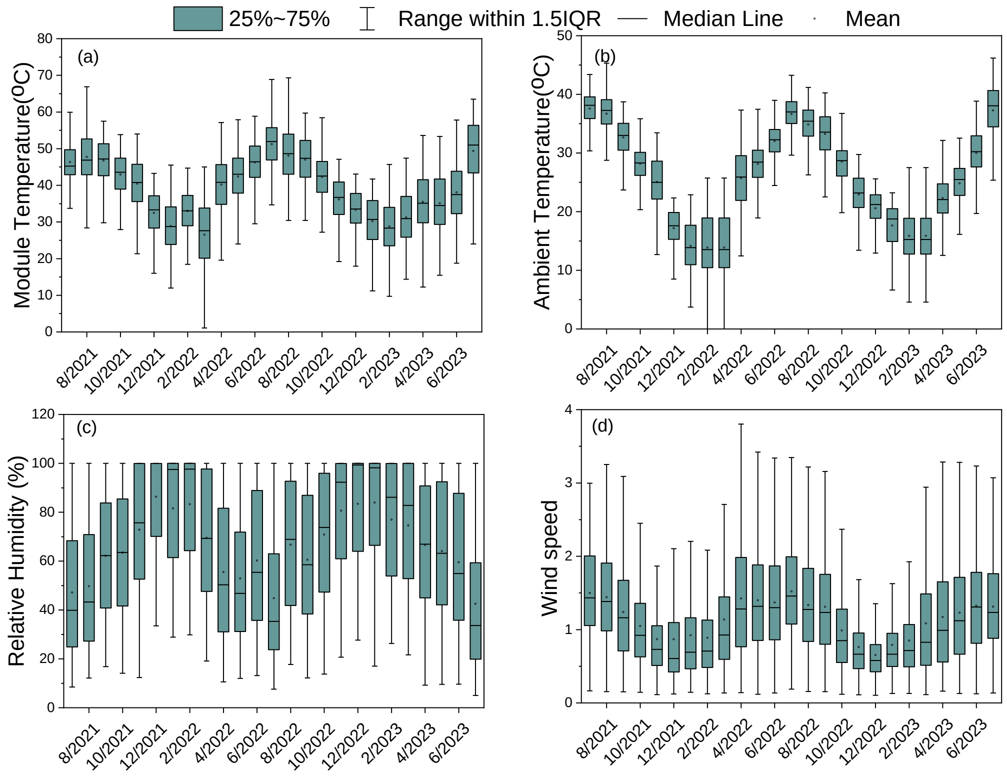

Figure 35:Statistical Analysis of weather variables from July 2021 until July 2023 at the testing site (a) Mini-Module Temperature, (b) Ambient Temperature, (c) Relative Humidity and (d) Wind Speed. The thick black line on each box represents the median value of the variables and the circle represents the mean value. The box represents the 25%-75% of the data and the data markers represent the outliers of the variables.

References

[1] E. Tubaldi, F. Freddi, and M. Barbato, “Measuring bias in structural response caused by ground motion scaling,” *Earthq. Eng. Struct. Dyn.*, vol. 45, no. 11, pp. 1743–1758, 2016, doi: 10.1002/eqe.

[2] A. Kyprianou, J. Giacomin, K. Worden, and M. Heidrich, “Differential evolution based identification of automotive hydraulic engine mount model parameters.,” *Proc. Inst. Mech. Eng. Part D J. Automob. Eng.*, vol. 214, no. 3, pp. 249–264, 2000.

[3] S.S. Rao, E*ngineering optimization: Theory and Practice, 3rd Edition, John. Wiley & Sons,*. 1996.

[4] S. Theocharides, G. Makrides, A. Livera, M. Theristis, P. Kaimakis, and G. E. Georghiou, “Day-ahead photovoltaic power production forecasting methodology based on machine learning and statistical post-processing,” *Appl. Energy*, vol. 268, no. December 2019, p. 115023, 2020, doi: 10.1016/j.apenergy.2020.115023.
